# Chaihu Guizhi Ganjiang Decoction Ameliorates Pancreatic Fibrosis via JNK/mTOR Signaling Pathway

**DOI:** 10.3389/fphar.2021.679557

**Published:** 2021-06-10

**Authors:** Lihua Cui, Caixia Li, Ye Shang, Dihua Li, Yuzhen Zhuo, Lei Yang, Naiqiang Cui, Yuhong Li, Shukun Zhang

**Affiliations:** ^1^ Tianjin Key Laboratory of Acute Abdomen Disease Associated Organ Injury and ITCWM Repair, Institute of Acute Abdominal Diseases of Integrated Traditional Chinese and Western Medicine, Tianjin Nankai Hospital, Nankai Clinical College, Tianjin Medical University, Tianjin, China; ^2^ Institute of TCM, Tianjin University of Traditional Chinese Medicine, Tianjin, China; ^3^ Department of Hepatobiliary and Pancreatic Surgery, Tianjin Nankai Hospital, Tianjin, China

**Keywords:** Chaihu Guizhi Ganjiang Decoction, chronic pancreatitis, pancreatic stellate cells, autophagy, JNK/mTOR

## Abstract

Pancreatic fibrosis is a pathological characteristic of chronic pancreatitis (CP) and pancreatic cancer. Chaihu Guizhi Ganjiang Decoction (CGGD) is a traditional Chinese medicine, which is widely used in the clinical treatment of digestive diseases. However, the potential anti-fibrosis mechanism of CGGD in treating CP remains unclear. Here, we conducted a series of experiments to examine the effect of CGGD on the CP rat model and primary isolated pancreatic stellate cells (PSCs). The results revealed that CGGD attenuated pancreatic damage, decreased collagen deposition, and inhibited PSC activation in the pancreas of CP rats. However, compared with the CP group, CGGD had no effect on body weight and serum amylase and lipase. In addition, CGGD suppressed autophagy by downregulating Atg5, Beclin-1, and LC3B and facilitated phosphorylation of mTOR and JNK in pancreatic tissues and PSCs. Moreover, the CGGD-containing serum also decreased LC3B or collagen I expression after rapamycin (mTOR inhibitor) or SP600125 (JNK inhibitor) treatment in PSCs. In conclusion, CGGD attenuated pancreatic fibrosis and PSC activation, possibly by suppressing autophagy of PSCs through the JNK/mTOR signaling pathway.

## Introduction

Chronic pancreatitis (CP) is a progressive pathological change in the pancreas, characterized by glandular atrophy and fibrosis, which leads to endocrine and exocrine dysfunction (Witt et al., 2007). To date, the clinical therapeutic intervention of CP is mainly symptomatic treatment including alleviation of pain, pancreatic enzyme supplementation, nutritional support therapy, and surgical therapy (Hart and Conwell, 2020). However, the underlying pathogenesis of CP is still unclear, and there is lack of specific therapeutic drugs for pancreatic fibrosis.

Pancreatic fibrosis is caused by the imbalance in the production and degradation of extracellular matrix (ECM). A large number of fibers replace the parenchyma of the pancreas, resulting in impaired pancreatic function and many other complications such as diabetes. Pancreatic stellate cells (PSCs) are the major effector cells in the initiation and development of pancreatic fibrogenesis (Erkan et al., 2012). In normal pancreatic tissues, PSCs only account for a small proportion in the pancreas and are inactivated. When pancreatic tissues are damaged, PSCs differentiate into myofibroblast-like cells, proliferate rapidly, and secrete a large amount of ECM including alpha-smooth muscle actin (α-SMA), collagen (COL), and fibronectin (FN). As a result, the pancreas parenchyma was replaced by the ECM, and pancreatic fibrosis occurred, thereby destroying the normal function. The studies reported that permanent activation of PSCs had been observed in CP patients and CP models (Zeng et al., 2019; Ramakrishnan et al., 2020). Therefore, aiming at inhibiting PSC activation might be a promising approach to alleviate pancreatic fibrosis.

Autophagy is a conservative lysosome-dependent process for maintaining cellular homeostasis, through which cytoplasmic components are packaged into specialized bilayer membranous structures called autophagosomes and degraded by lysosomes for recycling of nutrients. The proteins such as Beclin-1, Atg5, LC3B, and P62 are involved in autophagy formation (Gonzalez et al., 2011). And mTOR is the key regulator of autophagy, which can be regulated by PI3K/AKT, AMPK, and MAPK signaling pathways (Tu et al., 2017; Xue et al., 2017). The mice with pancreas-specific deletion of Atg5 showed symptoms similar to those of human CP (Diakopoulos et al., 2015). Autophagic alanine secretion in PSCs supports pancreatic tumor metabolism (Sousa et al., 2016). In 2017, Endo et al. reported that autophagy participated in PSC activation (Endo et al., 2017). Enhanced autophagy facilitates PSC activation and pancreatic fibrogenesis in CP (Li et al., 2018b). Our previous studies confirmed that autophagy enhanced gradually when the PSC culture time prolonged. Inhibited autophagy could reduce the accumulation of ECM proteins (Li et al., 2018b). We also found that autophagy activity was significantly increased in rat CP (Cui et al., 2019). These studies indicated that targeting to suppress PSC autophagy may be an effective approach for inhibiting PSC activation and pancreatic fibrosis.

According to the traditional Chinese medicine theory, CP can be attributed to the shao-yang and yangming signs of abdominal pain and rib-side distention and is related to the dysfunction of the liver, gallbladder, and spleen. Chaihu Guizhi Ganjiang Decoction (CGGD) is a traditional Chinese medicine formula that was first described by Zhongjing Zhang in “Treatise on Febrile Diseases Caused by Cold (Shanghan Lun)” and was used to treat shao-yang and yangming symptoms. CGGD is composed of seven herbs including *Bupleurum chinense DC., Neolitsea cassia (L.) Kosterm., Zingiber officinale Roscoe, Trichosanthes kirilowii Maxim., Scutellaria baicalensis Georgi, Ostrea gigas Thunberg, and Glycyrrhiza uralensis Fisch. ex DC.*, which has been widely used in the clinical treatment of digestive, respiratory, and cardiovascular diseases in traditional Chinese medicine (Chen et al., 1995; Itoh et al., 1996). In Chinese hospitals, CGGD was also used to treat patients with pancreatic cancer (Ma and Chen, 2017) and CP (Ding et al., 2017). It was found that it could improve the clinical symptoms of patients, but the underlying mechanism remains unclear. Modern medical studies have shown that saikosaponin A and saikosaponin D from *Bupleurum chinense* and baicalin from *Scutellaria baicalensis* could inhibit the activation of PSCs and ameliorate pancreatic fibrosis (Cui et al., 2019; Cui et al., 2020; Fan et al., 2020). The active components in *Glycyrrhiza uralensis, Bupleurum chinense, and Scutellaria baicalensis* also exhibited regulation of autophagy (Hong et al., 2018; Cui et al., 2019; Li et al., 2020). The aim of the present study was to explore the effect of CGGD on pancreatic fibrosis induced by dibutyltin dichloride (DBTC) and the mechanism of CGGD for inhibiting pancreatic fibrosis by suppressing PSC autophagy.

## Materials and Methods

### Materials and Reagents

CGGD full ingredient formula granules were purchased from Beijing Tcmages Pharmaceutical Co., Ltd. (Beijing, China). CGGD consists of seven herbs including *Bupleurum chinense DC.* (batch number: 18013961), *Neolitsea cassia (L.) Kosterm.* (batch number: 18000871), *Zingiber officinale Roscoe* (batch number: 1809811), *Trichosanthes kirilowii Maxim.* (batch number: 18012241), *Scutellaria baicalensis Georgi* (batch number: 1809691), *Ostrea gigas Thunberg* (batch number: 18017841), and *Glycyrrhiza uralensis Fisch. ex DC.* (batch number: 18010991) according to “shang han lun,” and the mixed proportion of respective herbs is illustrated in Table 1. The voucher specimens were deposited in the Institute of Acute Abdominal Diseases of Integrated Traditional Chinese and Western Medicine, Tianjin Nankai Hospital. Saikosaponin A, saikosaponin D (identified for *Bupleurum chinense DC.*), cinnamaldehyde, cinnamic acid (identified for *Neolitsea cassia (L.) Kosterm.*), 6-gingerol, 8-gingerol, 10-gingerol, zingerone (identified for *Zingiber officinale Roscoe*), γ-aminobutyric acid (identified for *Trichosanthes kirilowii Maxim.*), baicalin, wogonoside, wogonin (identified for *Scutellaria baicalensis Georgi*), liquiritin, liquiritin apioside, isoliquiritin apioside, and glycyrrhizic acid (identified for *Glycyrrhiza uralensis Fisch. ex DC.*) (the purities of all standards were higher than 98% by HPLC analysis) were purchased from Shanghai Yuanye Bio-Technology Co., Ltd. (Shanghai, China). Anti-α-SMA (A5228) was obtained from Sigma-Aldrich Chemical Co. (St. Louis, MO, United States). Anti-collagen I (ABT123) was obtained from Millipore (Billerica, MA, United States). Anti-LC3B was obtained from Novus Biologicals (Littleton, CO, United States). Glyceraldehyde 3-phosphate dehydrogenase (GAPDH, 5174), Atg5 (12994), Beclin-1 (3495), P62 (5114), *p*-ERK1/2 (4370s), *p*-P38 (9211s), *p*-JNK (9255s), *p*-AKT (4060s), *p*-AMPK (2535), and *p*-mTOR (2971) antibodies were obtained from Cell Signaling Technology (Beverly, MA, United States). Fibronectin (ab2413), MMP2 (ab37150), and TIMP2 (ab1828) antibodies were purchased from Abcam (Cambridge, MA, United States). Rapamycin was purchased from Selleck Chemicals (Shanghai, China). SP600125 was provided by MCE (Shanghai, China).

**TABLE 1:** Contents of CGGD.

Scientific name	Chinese name	English name	Crude drug (g)	Granules (g)
*Bupleurum chinense* DC.	Chai hu	Bupleuri Radix	15	1.50
*Neolitsea cassia* (L.) Kosterm.	Gui zhi	Cinnamomi Ramulus	12	0.60
*Zingiber officinale* Roscoe	Gan jiang	Zingiberis Rhizoma	6	0.30
*Trichosanthes kirilowii* Maxim.	Tian hua fen	Trichosanthis Radix	12	1.20
*Scutellaria baicalensis* Georgi.	Huang qin	Scutellariae Radix	9	0.90
*Ostrea gigas* Thunberg	Mu li	Ostreae concha	20	0.67
*Glycyrrhiza uralensis* Fisch. ex DC.	Gan cao	Glycyrrhizae Radix et Rhizoma	3	0.60

### HPLC Analysis of Chaihu Guizhi Ganjiang Decoction

The CGGD formula granules were pulverized into homogenous powder (through a 65-mesh sieve), and then the powder (0.1 g) was extracted with methanol (1:100 g/mL) in an ultrasonic water bath at 40°C for 30 min. The solution was centrifuged at 7,000 rpm for 10 min and then filtered with a 0.22 μm filter. Aliquot (10 μL) of the solution was injected into HPLC-DAD (Thermo Scientific Ultimate 3000) for analysis. An Agilent Eclipse Plus C18 column (4.6 mm × 250 mm, 5 μm) was used at a column temperature of 30°C. The sample was eluted with the mobile phase of A (0.1% (v/v) formic acid) and B (acetonitrile) under the following gradient program: 5–10% B for 0–2 min, 10–14% B for 2–5 min, 14–18% B for 5–8 min, 18–20% B for 8–10 min, 20–21% B for 10–13 min, 21–25% B for 13–17 min, 25–25% B for 17–30 min, 25–38% B for 30–33 min, 38–40% B for 33–43 min, 40–50% B for 43–50 min, 50% B for 50–55 min, 50–95% B for 55–60 min, and 95% B for 60–62 min. The analytes were detected at the wavelength of 258 nm. The flow rate was 1.0 ml/min. Between two analytes, the column was re-equilibrated with 15% B for 10 min.

### Animals and Grouping

Thirty male Wistar rats (weight: 170–190 g) were purchased from Vital River Laboratory Animal Technology Co., Ltd. (Beijing, China). The rats were raised under specific pathogen-free conditions: 12 h light/dark cycle at 22–24°C. The animals were randomly divided into three groups (*n* = 10 per group): control group, CP group, and CP treated with CGGD group. All experiments were conducted in accordance with the Chinese Guide for the Care and Use of Laboratory Animals as well as the methods for the management of experimental animals. The animal experiments were approved by the Animal Ethics Committee of Tianjin Nankai Hospital (Approval No. NKYY-DWLL-2019-003).

### Chronic Pancreatitis Rat Models’ Induction and Treatment

The CP model was induced by caudal vein injection of dibutyltin dichloride (DBTC) according to previously published protocols (Zhang et al., 2017). Briefly, DBTC (Sigma-Aldrich, China) was first dissolved in Part Ⅰ (absolute ethyl alcohol) and then mixed with Part Ⅱ (glycerol) and Part Ⅲ (DMSO). The proportion of three solutions was 1:2:2. The CP group and CGGD group were injected with DBTC solution at a dose of 7 mg/kg (Inoue et al., 2002). The control group was treated with the same volume of solvent (ethanol:glycerol:DMSO, 1:2:2). One day after DBTC injection, the CGGD group rats received CGGD (1.44 g/kg body weight/dose, 144 mg/ml concentration) by gavage once daily, according to the body weight conversion between rats and humans, that is, all the granules’ weight 5.77 g/60 kg (human body weight) × 15 (conversion coefficient for humans and rats) = 1.44 g/kg per day. The rats in the control group and CP group were given the same volume of distilled water by gavage once daily. After 4 weeks, the animals were anesthetized and abdominal aortic blood was taken for serum collection, and the serum was stored at −80°C until use. The pancreatic tissues were harvested, and part of each was immediately stored at −80°C, while the other part of each was fixed in neutral phosphate formaldehyde for further experiments.

### Histological Examinations

The formaldehyde-fixed pancreatic tissues were embedded in paraffin wax and cut into 5 μm sections. For morphological assessment, tissue sections were stained with hematoxylin and eosin (H&E). The results of histological changes were evaluated by two pathologists and scored on four indicators, including inflammation, abnormal architecture, glandular atrophy, and fibrosis, in accordance with previous reports (Niina et al., 2014). The collagen fiber deposition was also assessed by sirius red staining (Zhao et al., 2010). The photographs were taken by Leica microscope DMI4000B.

### Amylase and Lipase Assay

The serum amylase and lipase were detected by kits (C016-1-1, A054-2-1) from Nanjing Jiang Bioengineering Institute (Nanjing, China). The experimental operation was carried out according to the instructions.

### Immunocytochemistry Staining

The pancreatic tissue sections were detected with Bioss Kit-SP0023 (Beijing, China) according to the manufacturer’s instructions. After blocking with goat serum, the sections were incubated with primary antibodies, α-SMA (dilution 1:500) and LC3B (1:300), overnight at 4°C. Then, the nucleus was counterstained with hematoxylin. After dehydration and mounting, the slides were photographed by a microscope.

### Isolation, Identification, and Culture of Pancreatic Stellate Cells

Pancreatic stellate cells (PSCs) were isolated from the rat pancreas as described previously (Li et al., 2018a). PSCs cultured for three generations were used for experiments.

### Preparation of Chaihu Guizhi Ganjiang Decoction–Containing Serum

Twenty male Wistar rats weighing 170–190 g were randomly divided into two groups, with 10 rats in each group. The CGGD group rats were orally administered for three consecutive days (1.44 g/kg body weight/dose), once a day. The control group rats were given distilled water. On the third day, the rats were anesthetized 2 h after final administration, the blood was collected from the abdominal aorta, and the serum was obtained by centrifugation at 3,000 rpm for 10 min. After 56°C water bath for 30 min, the serum was filtered with a 0.22 μm cellulose acetate membrane and stored at −80°C for use.

### RNA Isolation and Real-Time PCR

Total RNA from pancreatic tissues was extracted by Trizol reagent (Promega, China). Then, the total RNA of each sample was reverse-transcribed into cDNA using the First Strand cDNA Synthesis Kit (cat. no. K1622; Thermo, United States). RT-PCR was performed using the DyNAmo Flash SYBR Green Kit (cat. no. F-415L; Thermo) and the Applied Biosystems 7500 Fast Real-Time PCR System (Thermo, Singapore). Primer sequences for RT-PCR analysis are listed in Table 2. The value of the target genes’ mRNA was expressed as a relative intensity normalized to the endogenous control GAPDH.

**TABLE 2:** Primer sequences used for RT-PCR analysis.

Gene	Forward sequence (5′-3′)	Reverse sequence (5′-3′)
α-SMA	AGG​GAG​TGA​TGG​TTG​GAA​TG	GAT​GAT​GCC​GTG​TTC​TAT​CG
COL I	GGATAGGGACTTGTGTGA	GCTGGAAGAGTGAAGAGG
FN	GAT​TCT​TCT​GGC​GTC​TGC​AC	GCC​CCG​GAA​CAT​GAG​GAT​AG
MMP2	AGA​AGG​CTG​TGT​TCT​TCG​CA	AAA​GGC​AGC​GTC​TAC​TTG​CT
TIMP2	ATTTATCTACACGGCCCC	CAA​GAA​CCA​TCA​CTT​CTC​TTG
Beclin-1	TGT​TTG​GAG​ATG​TTG​GAG​CA	ATG​GAA​GGT​CGC​ATT​GAA​GA
Atg5	TGA​AGG​AAG​TTG​TCT​GGA​TAG	AAG​TCT​GTC​CTT​CCG​CAG​TC
LC3B	CGG​AGC​TTC​GAA​CAA​AGA​GTG	CTT​GGT​CTT​GTC​CAG​GAC​GG
GAPDH	AGA​TGG​TGA​AGG​TCG​GTG​TG	CTG​GAA​GAT​GGT​GAT​GGG​TT

### Western Blot Analysis

Total protein was extracted from pancreatic tissues using RIPA lysis buffer adding protease inhibitor and phosphatase inhibitor (Millipore, United States). The protein concentrations were measured by the BCA Protein Assay Kit (Pierce, United States). Protein samples were separated by SDS-PAGE and then electro-transferred to the polyvinylidene fluoride membrane (Millipore, United States). The membranes were blocked with 5% non-fat milk and probed with primary antibodies at 4°C overnight and subsequently secondary antibodies (1:5,000) for 1.5 h at room temperature. The protein bands on membranes were visualized by the Chemiluminescent Substrate Kit (Pierce, United States) and quantified using the Chemidoc XRS System (Bio-Rad, CA).

### Immunofluorescence Staining

A total of 5×10^4^ cells/well were seeded in a six-well plate. After cells adhered to the plate, PSCs were incubated with control serum (CONs) and CGGD serum (CGGDs) for 24 h, or pretreated with 10 μM rapamycin or 20 μM SP600125 separately for 1 h, and then combined with CONs or CGGDs for 24 h. The cells were fixed with neutral formalin and followed by staining with α-SMA (dilution 1:500) and LC3B (dilution 1:300). The nucleus was counterstained with DAPI. The images were captured using fluorescent microscopy.

### Statistical Analysis

All data are presented as mean ± standard derivation (SD). Statistical analyses were performed using one-way ANOVA or two-way ANOVA and Tukey’s test analysis by Prism software (GraphPad). A value of *p* < 0.05 was considered to be statistically significant.

## Results

### HPLC Chromatograms of Chaihu Guizhi Ganjiang Decoction

**FIGURE 1:**
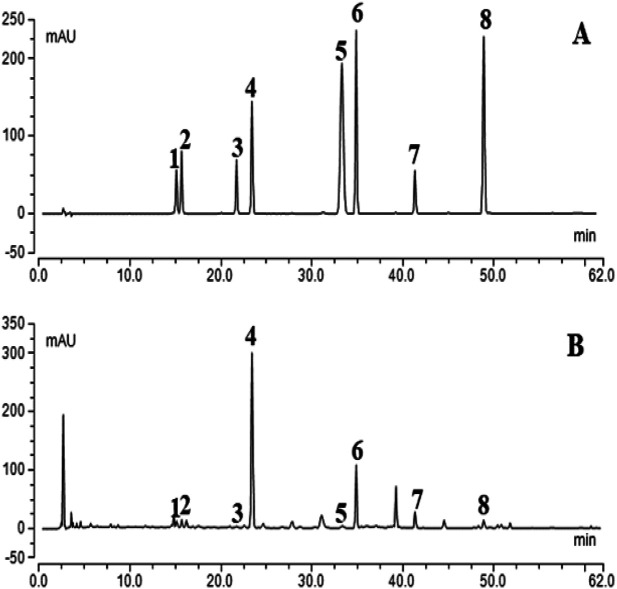
Typical chromatograms of mixed standard compounds **(A)** and CGGD **(B)**. (1) liquiritin apioside, (2) liquiritin, (3) isoliquiritin apioside, (4) baicalin, (5) cinnamic acid, (6) wogonoside, (7) glycyrrhizic acid, and (8) wogonin.

According to the Pharmacopoeia of China, one or more standard ingredients were selected for each herbal medicine. The chromatograms of eight mixed standards and the extracts of CGGD formula granules are illustrated in Figure 1. The result showed that the contents of the investigated analytes were as follows: 1.94 ± 0.02 mg/g of liquiritin apioside, 1.67 ± 0.01 mg/g of liquiritin, 0.46 ± 0.01 mg/g of isoliquiritin apioside, 22.34 ± 0.89 mg/g of baicalin, 0.26 ± 0.00 mg/g of cinnamic acid, 4.42 ± 0.17 mg/g of wogonoside, 4.91 ± 0.19 mg/g of glycyrrhizic acid, and 0.61 ± 0.02 mg/g of wogonin (*n* = 3). But the contents of saikosaponin A, saikosaponin D, γ-aminobutyric acid, cinnamaldehyde, 6-gingerol, 8-gingerol, 10-gingerol, and zingerone were not detected under the condition.

### The Effects of Chaihu Guizhi Ganjiang Decoction on the Body Weight, Serum Amylase and Lipase, and Morphological Changes in Rats With Chronic Pancreatitis

**FIGURE 2:**
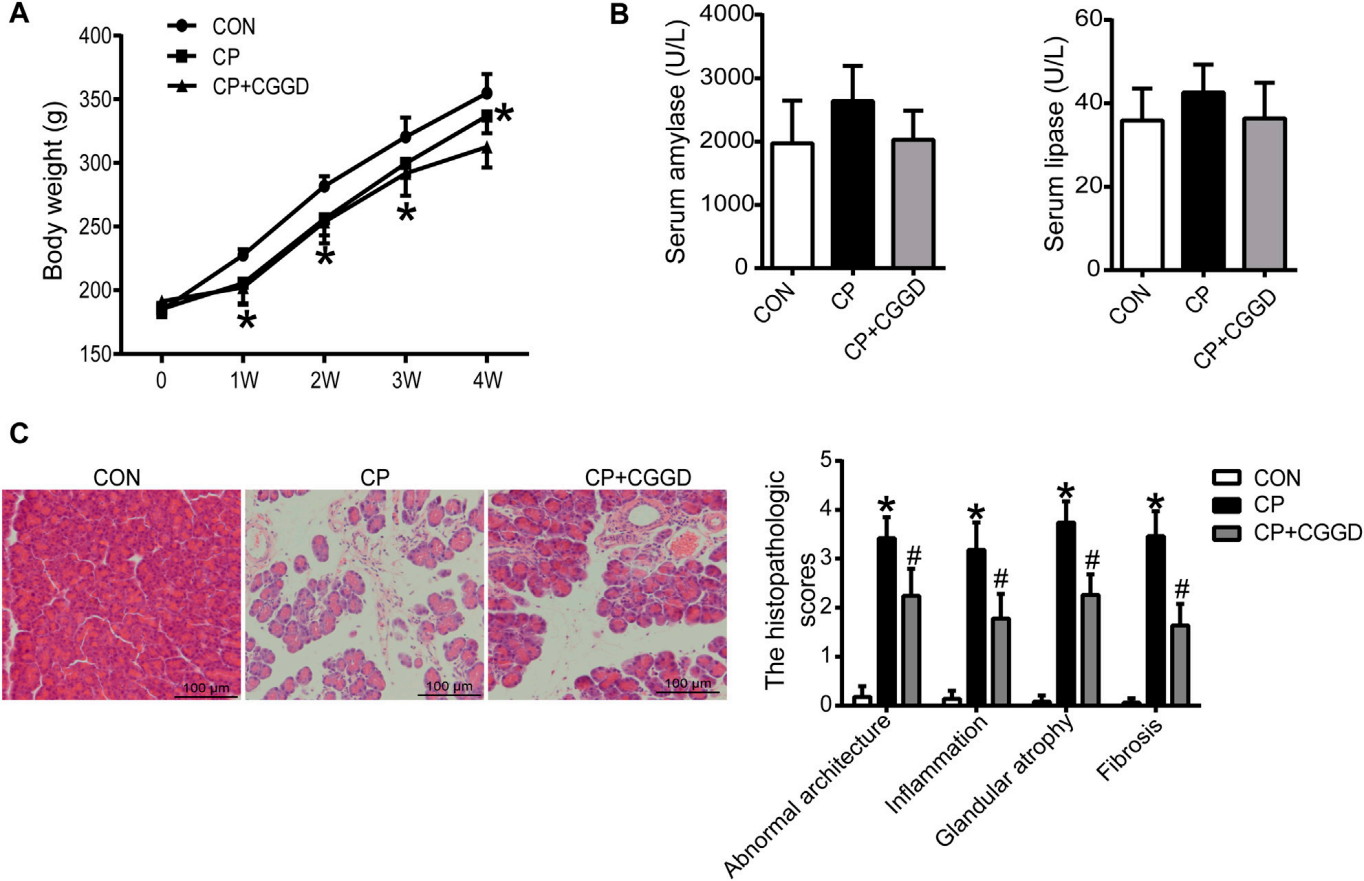
Effects of CGGD on the body weight, serum amylase and lipase, and histological changes in CP rats induced by DBTC. **(A)** Body weight at different time points. **(B)** Serum amylase and lipase detected by ELISA. **(C)** Pancreatic morphological changes observed by H&E staining and scored by a pathologist (original magnification, × 200). *n* = 7–10, ^*^ *p* < 0.05 compared with the control group; ^#^ *p* < 0.05 compared with the CP group.

Throughout the experiment, the body weight of all rats was monitored. In the first 7 days after injection of DBTC, most rats exhibited significant illness including ruffled fur, loss of activity, and jaundice in their tails. But the rat activity in the CP + CGGD group was a bit better than that of CP rats. Thereafter, these features improved gradually following the recovery time, and the common phenotypic characteristics in CP and CP + CGGD groups showed no differences. As shown in Figure 2A, in the CP group, the rat growth rate slowed down during the experimental period, and the body weight was decreased significantly compared with the control ones. However, the change caused by CP was not restored by treatment with CGGD. The serum amylase and lipase reflect the secretory function of the pancreas. In the three groups, they were similar, respectively, with no statistical difference found (Figure 2B). Light microscopic investigations showed that control group rats had a normal histological architecture of the pancreas. In the CP group, DBTC induced severe histological changes, including inflammatory cell infiltrates, abnormal architecture, glandular atrophy, and fibrosis (Figure 2C). Treatment with CGGD significantly attenuated the markers of pancreatic damage compared with the CP group.

### Chaihu Guizhi Ganjiang Decoction Decreased Collagen Deposition and PSC Activation in Pancreas

**FIGURE 3:**
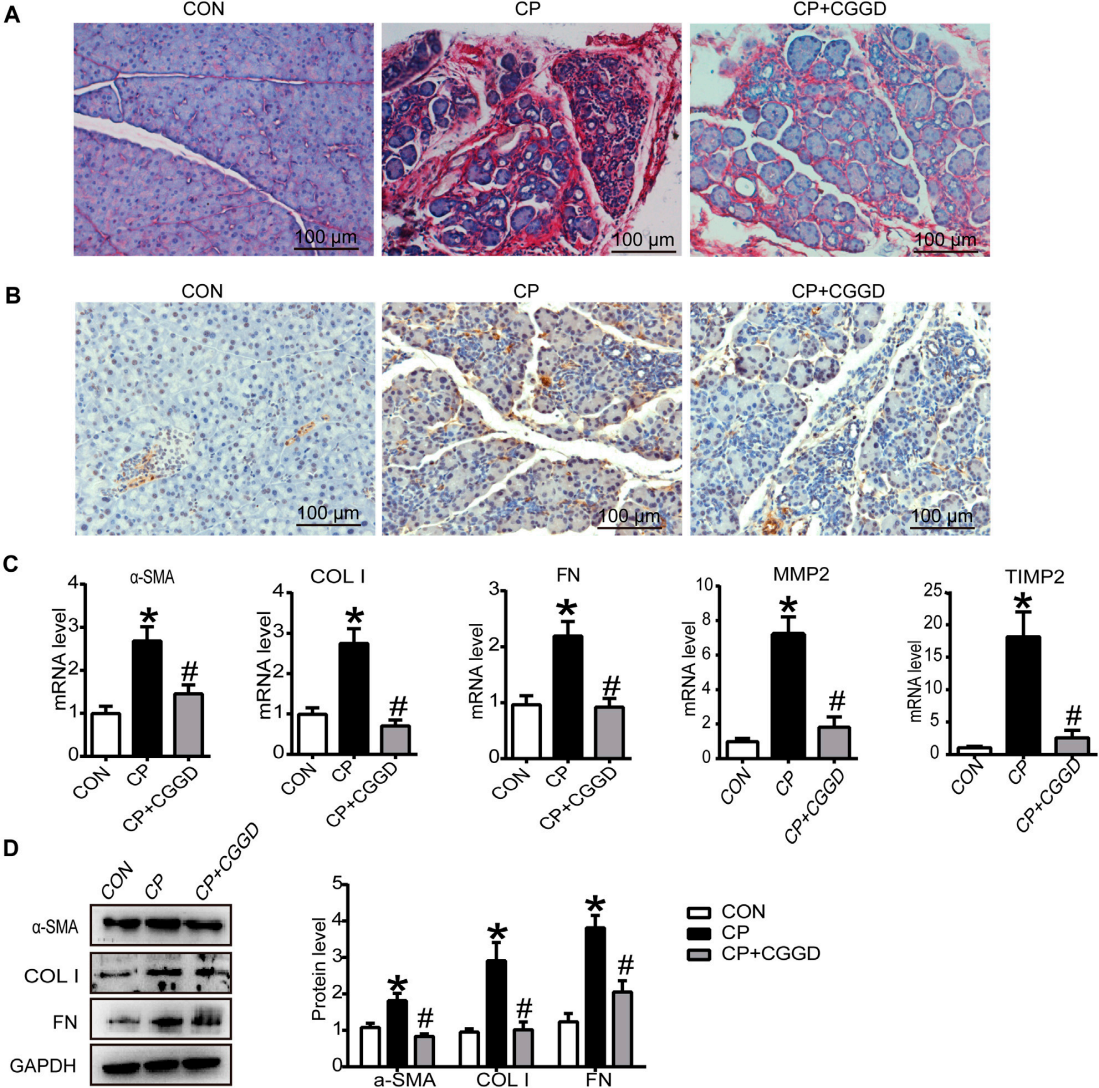
Effects of CGGD on collagen deposition and PSC activation in the pancreas. **(A)** Sirius red staining for collagen deposition in the pancreas (original magnification, ×200). **(B)** Immunohistochemistry staining of α-SMA (brown staining) in pancreatic tissues. Hematoxylin was used to counterstain nuclei (original magnification, ×200). **(C)** qRT-PCR analysis of α-SMA, COL I, FN, MMP2, and TIMP2. **(D)** Western blot analysis of α-SMA, COL I, and FN. Data are expressed as mean ± SD (n = 3). ^*^ *p* < 0.05 compared with the control group; ^#^ *p* < 0.05 compared with the CP group.

Sirius red staining displayed the collagen deposition in the pancreas. As shown in Figure 3A, there were more red-stained areas in CP rats, while treatment with CGGD significantly decreased the COL content (sirius red–positive area). Activated PSCs specifically express α-SMA and collagen, leading to pancreatic fibrosis. As shown in Figure 3B, the activation marker of PSC, α-SMA, was increased significantly in the CP group compared with the control group. CGGD treatment reduced the α-SMA expression compared to the CP ones. The mRNA levels of α-SMA, COL I, FN, MMP2, and TIMP2 in CP were all increased, while CGGD treatment decreased the expression of these markers significantly (Figure 3C). The changes in protein levels of α-SMA, COL I, and FN were consistent with the mRNA levels (Figure 3D).

### Chaihu Guizhi Ganjiang Decoction Suppressed Pancreatic Autophagy in Chronic Pancreatitis Rats

**FIGURE 4:**
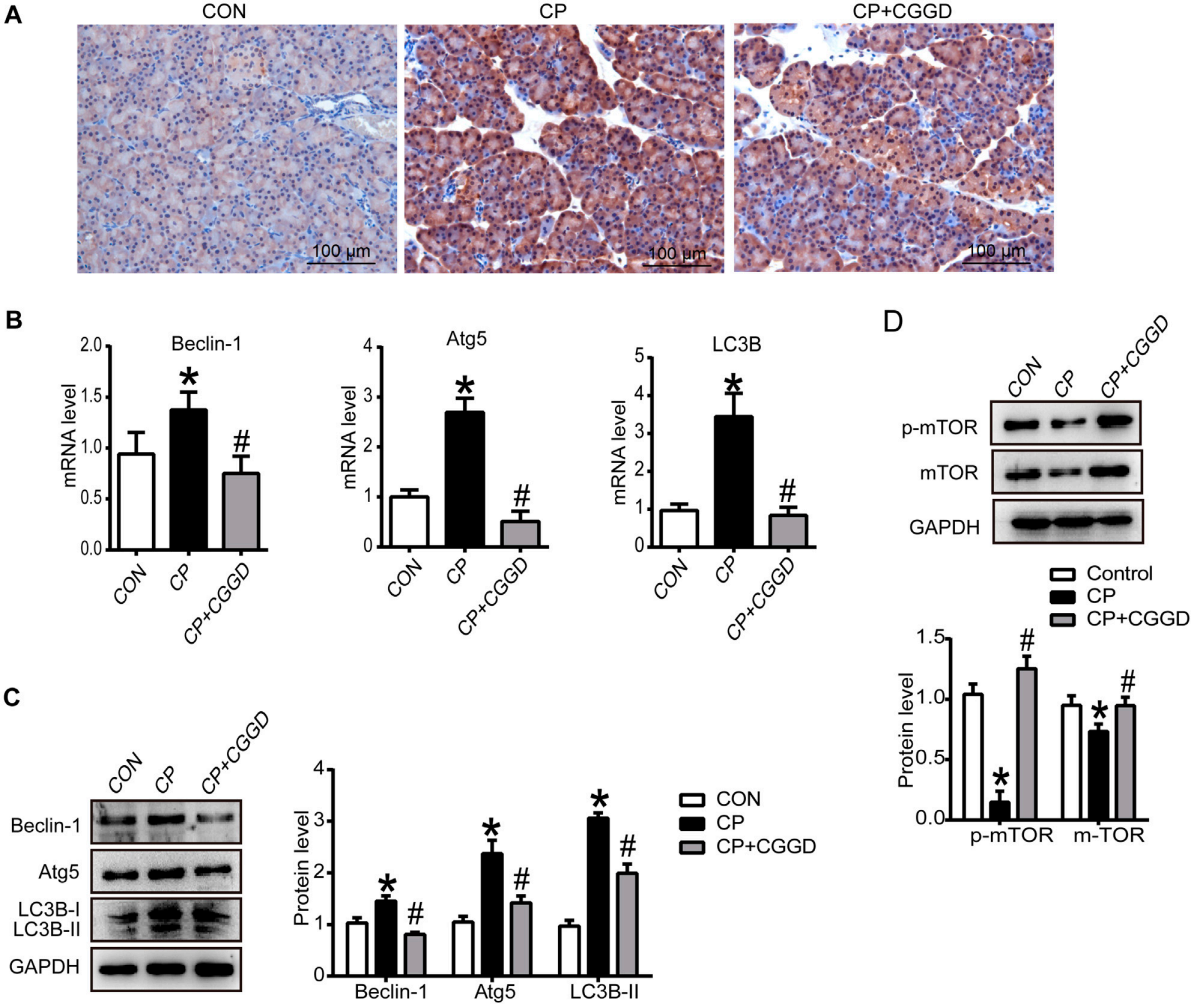
Influence of CGGD on autophagy and the mTOR pathway in the pancreas. **(A)** Immunohistochemistry staining of LC3B (brown staining) in pancreatic tissues. Hematoxylin was used to counterstain nuclei (original magnification, ×200). **(B)** mRNA levels of Beclin-1, Atg5, and LC3B in the pancreas detected by qRT-PCR. **(C)** Western blot analysis of Beclin-1, Atg5, and LC3B-I/II in the pancreas. **(D)** Western blot analysis of *p*-mTOR and mTOR in the pancreas. GAPDH was used as the loading control. Data are expressed as mean ± SD (*n* = 3). *p < 0.05 vs. control; #*p* < 0.05 vs. CP.

It is known that autophagy participates in PSC activation. Immunohistochemistry staining showed that the LC3B expression level was upregulated in the CP group compared with the control group, and the level of LC3B was downregulated markedly by CGGD (Figure 4A). Meanwhile, the mRNA expression levels of LC3B, Atg5, and Beclin-1 were significantly increased in the CP group, and these levels were suppressed in the CP + CGGD group (Figure 4B). Moreover, western blotting analysis demonstrated that the expressions of Atg5, Beclin-1, and LC3B were basically consistent with the corresponding RT-PCR results (Figure 4C).

The mTOR expression in pancreatic tissues was detected by western blot. The protein levels of mTOR and *p*-mTOR were suppressed in the CP group, and CGGD treatment increased these protein expressions (Figure 4D).

### Chaihu Guizhi Ganjiang Decoction–Containing Serum Inhibited Pancreatic Stellate Cell Activation and Autophagy

**FIGURE 5:**
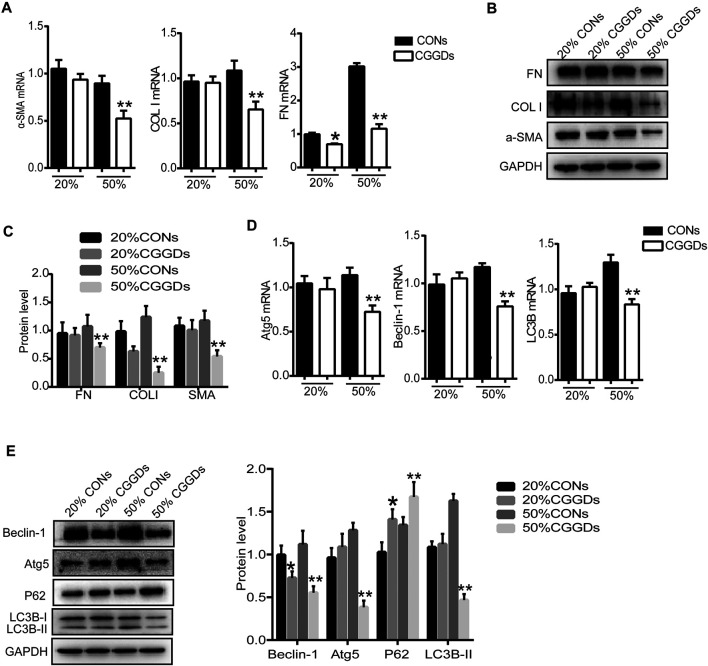
CGGD-containing serum (CGGDs) inhibited PSC activation and autophagy. PSCs were treated with 20% or 50% control serum (CONs) or CGGDs, respectively, for 24 h. **(A)** RT-PCR analysis of α-SMA, COL I, and FN. **(B)** Western blot analysis of α-SMA, COL I, and FN. **(C)** Densitometry analysis of the western blot bands of (B). **(D)** mRNA levels of Atg5, Beclin-1, and LC3B detected by RT-PCR. **(E)** Western blot analysis of Beclin-1, Atg5, P62, and LC3B. The data are expressed as fold changes over the value of the 20% CONs group. The data are expressed as the mean ± SD of three independent experiments. *p < 0.05 vs. the 20% CONs group; **p < 0.05 vs. the 50% CONs group.

In order to determine the effect of CGGDs on PSCs, different proportions of control serum (CONs) and CGGDs were added to PSCs for 24 h, and the target markers were detected. The mRNA level of FN was decreased by 20% CGGDs, but the levels of α-SMA and COL I showed no significant change compared with the 20% CONs (Figure 5A). The mRNA and protein levels of α-SMA, COL I, and FN, were all decreased significantly by 50% CGGDs compared with the 50% CONs (Figures 5A–C). These results showed that CGGDs inhibited PSC activation in a dose-dependent manner.

Compared with the 50% CONs, the mRNA expressions of Atg5, Beclin-1, and LC3B were all downregulated significantly by 50% CGGDs (Figure 5D). The western blot results were similar to the mRNA results. The P62 expression level was increased in the CGGDs group compared with the CONs group, indicating the lower level of autophagy (Figure 5E). The above results reveal a suppressive effect of CGGDs on PSC autophagy and activation.

### Chaihu Guizhi Ganjiang Decoction Serum Inhibited Pancreatic Stellate Cell Autophagy and Activation Through JNK/mTOR Pathway

**FIGURE 6:**
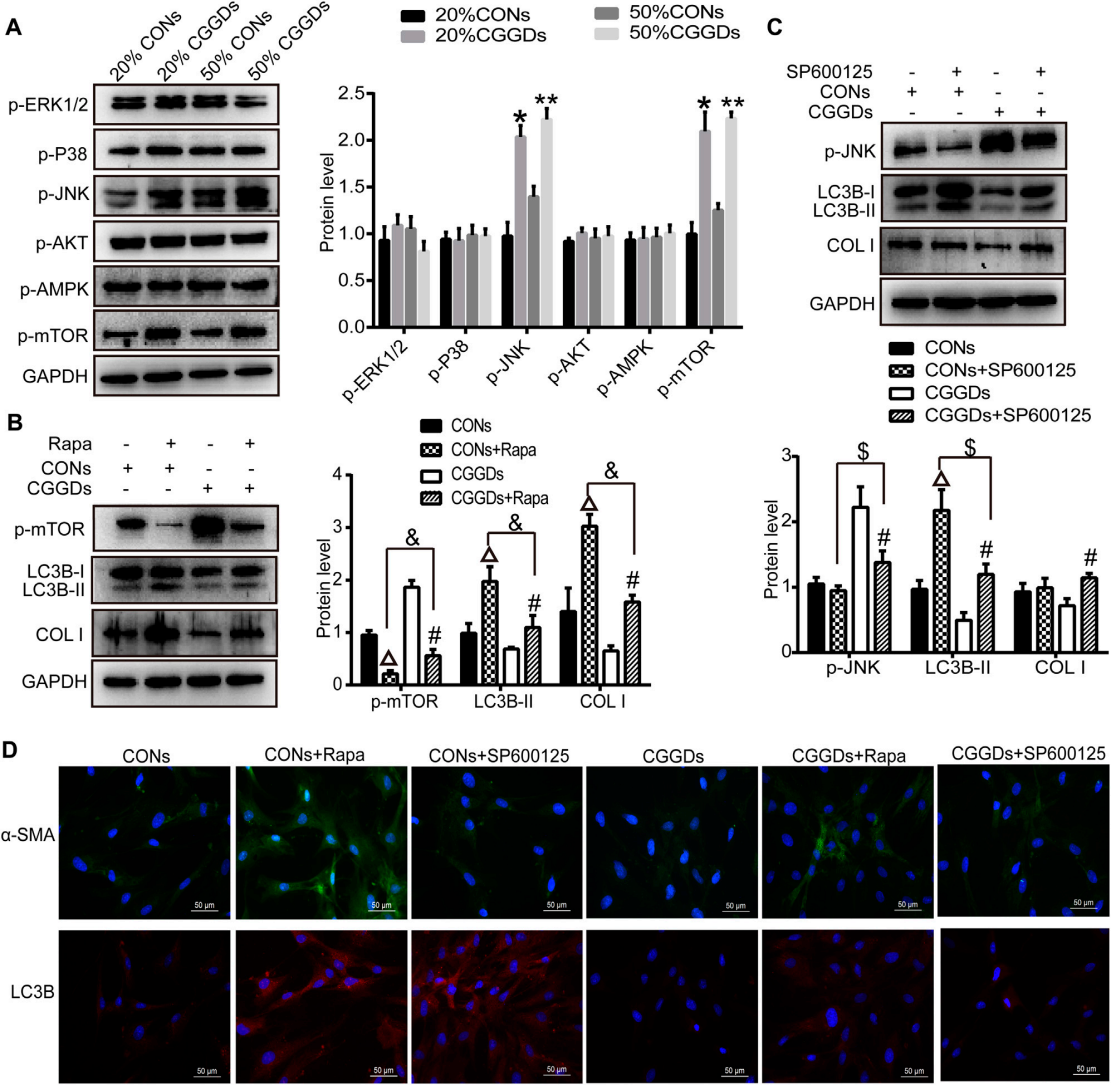
Effect of CGGDs on PSC autophagy and activation through the JNK/mTOR pathway. **(A)** Western blot analysis of *p*-ERK1/2, *p*-P38, *p*-JNK, *p*-AKT, *p*-AMPK, and *p*-mTOR after PSCs were treated with 20% or 50% CONs or CGGDs, respectively, for 24 h. **(B)** Protein levels of *p*-mTOR, LC3B-I/II, and COL I in PSCs detected after pretreatment with rapamycin (Rapa, an mTOR inhibitor) for 1 h followed by treatment with 50% CONs or 50% CGGDs for 24 h. **(C)** Expression levels of *p*-JNK, LC3B-I/II, and COL I in PSCs measured by western blot after treatment with SP600125 (a JNK inhibitor) for 1 h and 50% CONs or 50% CGGDs for 24 h. **(D)** Immunofluorescence staining used to detect the expression levels of α-SMA (green) and LC3B (red) after PSCs were pretreated with Rapa or SP600125 for 1 h followed by treatment with 50% CONs or 50% CGGDs for 24 h. The nuclei were stained with DAPI (blue) (original magnification: ×200). The data are expressed as the mean ± SD of three independent experiments. *p < 0.05 vs. the 20% CONs group, **p < 0.05 vs. the 50% CONs group, △*p* < 0.05 vs. the CONs group, #*p* < 0.05 vs. the CGGDs group, *p* < 0.05 vs. the CONs + Rapa group, and $*p* < 0.05 vs. the CONs + SP600125 group.

mTOR is a downstream target for MAPK, PI3K/AKT, and AMPK, the activation of which suppresses autophagy. After PSCs were treated with CGGDs for 24 h, the levels of *p*-mTOR and *p*-JNK were markedly increased in the presence of 20% and 50% CGGDs compared with the corresponding CONs. However, the levels of *p*-ERK1/2, *p*-P38, *p*-AKT, and *p*-AMPK were not changed in the absence or presence of CGGDs (Figure 6A).

Furthermore, the mTOR inhibitor rapamycin and JNK inhibitor SP600125 were applied to evaluate the role of mTOR and JNK in CGGDs-induced inhibition of PSC activation. PSCs were pretreated with or without rapamycin for 1 h and then incubated with 50% CGGDs or 50% CONs for 24 h. Western blot results indicated that rapamycin suppressed the level of *p*-mTOR in CONs + Rapa– and CGGDs + Rapa–treated cells compared with CONs and CGGDs ones, respectively. The expression of *p*-mTOR was much higher in the CGGDs + Rapa group than the CONs + Rapa group. Moreover, LC3B-II and COL I were both increased in CGGDs/CONs + Rapa groups, and the increased expression caused by Rapa (CONs + Rapa) can be decreased by CGGDs (CGGDs + Rapa) significantly (Figure 6B). The immunofluorescence staining showed similar results to Figure 6B, with increasing α-SMA and LC3B in CONs + Rapa and CGGDs + Rapa groups, and CGGDs suppressed the levels of α-SMA and LC3B that were elevated by Rapa (Figure 6D). These results indicated that CGGDs could inhibit PSC autophagy and activation by mTOR.

On pretreatment with the JNK inhibitor SP600125, the expression of *p*-JNK was reduced significantly, while the level of LC3B was increased significantly (CONs + SP600125 vs. CONs and CGGDs + SP600125 vs. CGGDs). CGGDs elevated the downregulated protein level of *p*-JNK induced by SP600125 and, meanwhile, reduced the high expression level of LC3B caused by SP600125 (CGGDs + SP600125 vs. CONs + SP600125) (Figure 6C). With the treatment of CGGDs, SP600125 exhibited promoting effect of COL I. But compared with CONs + SP600125 treatment, CGGDs + SP600125 treatment showed no influence on the COL I protein expression. Moreover, as shown in Figure 6D, the α-SMA expression was not different in CONs, CONs + SP600125, and CGGDs + SP600125 groups. However, the level of α-SMA in the CGGDs + SP600125 group was higher than that in the CGGDs group. The immunofluorescence results of LC3B were consistent with the western blot ones. These results revealed CGGDs could suppress PSC autophagy by JNK. Furthermore, the inhibitory effect of CGGDs on PSC activation is partly through the JNK pathway.

## Discussion

**FIGURE 7:**
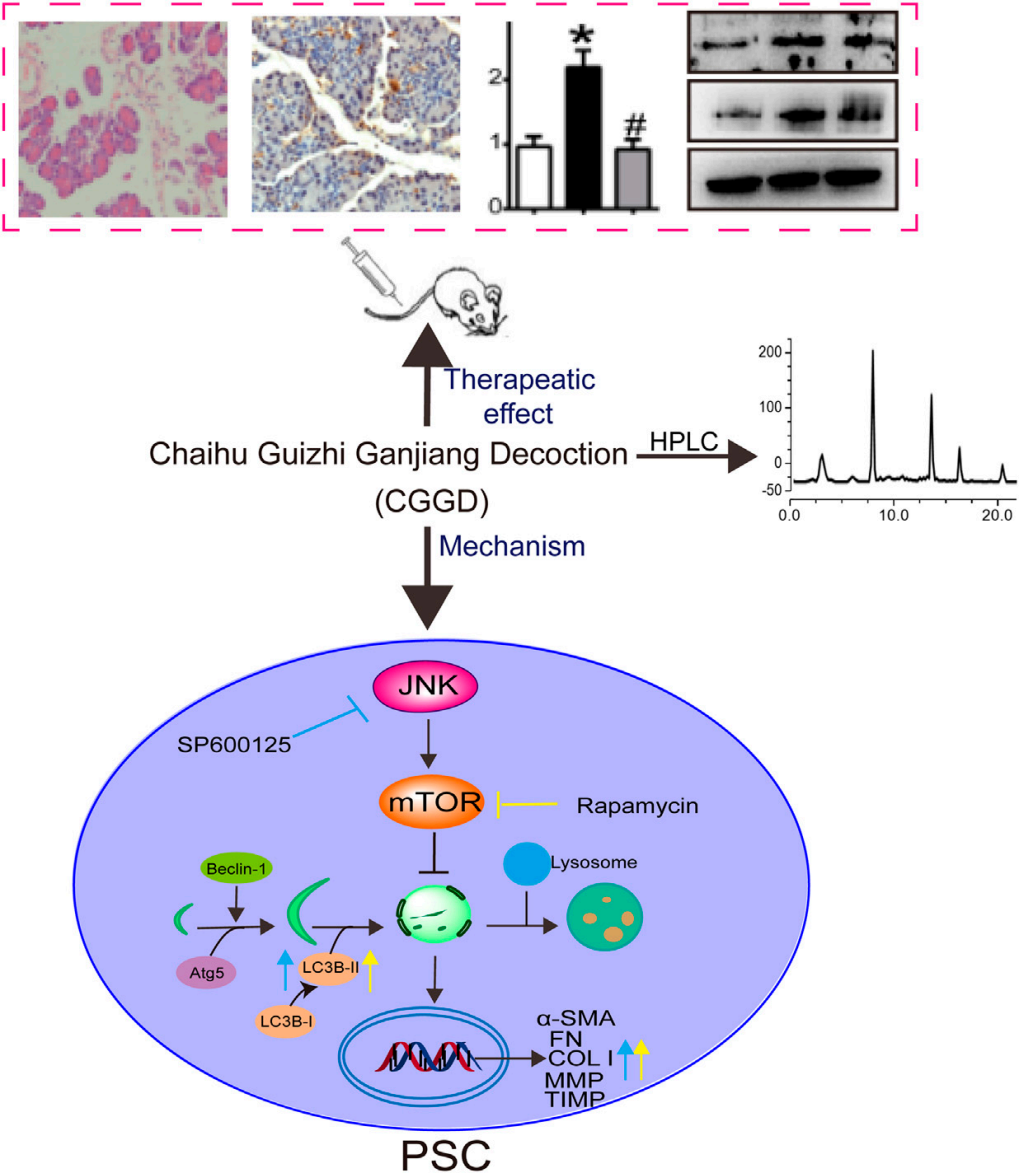
Graphical abstract of CGGD-ameliorated pancreatic fibrosis through deactivation of PSC by inhibiting autophagy via the JNK/mTOR pathway.

In this paper, we investigated the anti-fibrotic effects of CGGD on DBTC-induced CP rats and isolated PSCs. The results revealed CGGD treatment decreased pancreatic damage, restrained PSC activation, and reduced the deposition of ECM. In addition, CGGD inhibited the autophagy in pancreatic tissues and isolated PSCs. Moreover, this study showed that CGGD suppressed the PSC activation and autophagy *via* the JNK/mTOR pathway. These findings demonstrate that CGGD ameliorated pancreatic fibrosis by inhibiting activation and autophagy of PSCs through the JNK/mTOR pathway (Figure 7).

Pancreatic fibrosis is a typical feature of the end stage of CP, with abundant ECM accumulation (Kirkegård et al., 2017). Accumulating studies have confirmed that massive proliferation and activation of PSCs is the key process in the development of pancreatic fibrosis (Omary et al., 2007). Inhibition of PSC activation could prevent pancreatic fibrosis (McCarroll et al., 2006). In recent years, increasing studies revealed the effectiveness of traditional Chinese herbal medicine in the treatment of diseases (C et al., 2019). Our previous studies indicated that Xiaochaihu Decoction (soup with *Bupleurum falcatum*) prevents the progression of CP by inhibiting the TGF-β/Smad pathway and promoting collagen degradation (Zhang et al., 2013; Zhang et al., 2017). Our research group focused on the role of *Bupleurum* prescription in the treatment of CP. CGGD was the modified formula of Xiaochaihu Decoction and has been widely applied in brain diseases and abdominal diseases in traditional Chinese medicine (Yang et al., 2009; Liu et al., 2019). In China, CGGD has already shown curative effects in treating patients with CP in clinics. Here, the application of CGGD decreased pancreatic damage, restrained the expression of PSC-activated markers, and reduced the deposition of ECM in the pancreas of CP. Moreover, CGGDs also inhibited the expressions of α-SMA, COL I, and FN in primary cultured PSCs.

Autophagy is related to various organ fibroses (Bai et al., 2017; Hosseinzadeh et al., 2018). PSC activation is closely related to autophagy and can promote pancreatic cancer growth and metastasis (Endo et al., 2017). Our previous study confirmed that autophagy participated in PSC activation and showed that inhibition of autophagy could suppress PSC activation and increase ECM degradation in primary cultured PSCs (Li et al., 2018b). Saikosaponins A and D, the active components of *Bupleurum chinense DC.*, inhibit the activation of PSCs by inhibiting autophagy (Cui et al., 2019; Cui et al., 2020). However, they were not detected by HPLC in CGGD formula granules in this study. Maybe saikosaponins A and D were lost in the formation of granules due to some reasons. There were studies that also reported liquiritin and glycyrrhizic acid, which are active components of *Glycyrrhiza uralensis*, could inhibit gastric cancer (Wei et al., 2017) and ameliorate ALI (Qu et al., 2019) *via* regulating autophagy. The active compounds of *Scutellaria baicalensis Georgi*, baicalin, wogonoside, and wogonin, also exhibited modulating ECM degradation and cell apoptosis by autophagy (Zhang et al., 2014; Hong et al., 2018; Li et al., 2020). In this study, CGGD decreased the upregulated levels of Beclin-1, Atg5, and LC3B in the pancreas of DBTC-induced CP and primary cultured PSCs, which is similar to that reported in the previous study (Li et al., 2018a). But the effects of the major compounds (baicalin, wogonoside, and glycyrrhizic acid) in CGGD on PSC autophagy need further verification.

mTOR is the key regulator of autophagy (Yu et al., 2018). In CP rats, the levels of *p*-mTOR and mTOR were inhibited, and these markers were upregulated after CGGD treatment. In the isolated PSCs, *p*-mTOR was increased significantly after treatment with CGGDs, and the increase was downregulated by the mTOR inhibitor rapamycin. The levels of LC3B-II, COL I, and α-SMA that were upregulated in Rapa + CONs were decreased in Rapa + CGGDs. These results indicated that CGGDs could suppress PSC autophagy and activation through mTOR.

Furthermore, we detected the upstream molecules that regulate mTOR after treatment with CGGDs. The results revealed that the levels of *p*-ERK1/2, *p*-P38, *p*-AKT, and *p*-AMPK showed no differences in CGGDs and CONs, but *p*-JNK was upregulated in CGGDs. Xu et al. (2018) demonstrated that JNK and ERK were involved in the PSC activation induced by TGF-β. And P38 MAPK mediates the activation of PSCs in the presence of ethanol or acetaldehyde (McCarroll et al., 2003). Moreover, JNK was involved in regulating autophagy and apoptosis (Tian et al., 2019). In this paper, *p*-JNK was increased after PSCs were treated with CGGDs for 24 h. The JNK inhibitor SP600125 was applied to verify the effect of JNK on PSC activation and autophagy after CGGDs treatment. The results showed SP600125 did not affect COL I and α-SMA expressions in PSCs treated with CONs, but these markers increased after PSC exposure to CGGDs, thus abolishing the downregulated levels of COL I and α-SMA by CGGDs. These are similar to the results of SP600125 that had no effect on the α-SMA expression of PSCs stimulated by ethanol (McCarroll et al., 2003) but are different from another study showing SP600125 blocked activation of PSCs induced by the platelet-derived growth factor (Masamune et al., 2004). After SP600125 treatment, LC3B-II was increased in PSCs and the upregulated level was also inhibited by CGGDs compared with SP600125 + CONs. The effect of SP600125 on autophagy in PSCs was different from that in other cells (Tian et al., 2019). JNK regulates a variety of biological processes, including cell apoptosis (Yue and López, 2020), and the influence of CGGDs on PSC apoptosis needs further discussion.

In this study, CGGD reduced pancreatic damage, prevented pancreatic fibrosis, but did not improve the CP-caused body weight loss of normal animals. In the future, prolonging the observing time or detecting more changes in other organs is more comprehensive. Some markers in several herbs (6-gingerol, 8-gingerol, 10-gingerol, zingerone for *Zingiber officinale Roscoe*; γ-aminobutyric acid for *Trichosanthes kirilowii Maxim.*) were not identified in CGGD formula granules by HPLC. Maybe the volatile oil was little or lost in the process of production and usage of granules. In the future, on combining the use with other sensitive methods such as gas chromatography and mass spectrometry, more ingredients could be identified. CGGD-containing serum was used in the *in vitro* study, revealing the inhibition of PSC autophagy and activation as a whole. However, the active ingredients in the serum remain unknown. With the application of metabolomics analysis and discovery of active ingredients of drugs, more and more targets will be revealed.

In summary, the present study provided evidence for the alleviating effects of CGGD on CP through multiple mechanisms, including reducing pancreatic damage, preventing pancreatic fibrosis, and inhibiting PSC autophagy and activation through the JNK/mTOR pathway. This study focused on PSC activation and autophagy in pancreatic fibrosis and to some extent explained the molecular mechanism of the inhibition of CP by CGGD, which provides a theoretical basis for further popularization and application of CGGD.

## Data Availability

The original contributions presented in the study are included in the article/Supplementary Material, and further inquiries can be directed to the corresponding authors.

## References

[B1] Bai F. Huang Q. Nie J. Lu S. Lu C. Zhu X. (2017). Trolline Ameliorates Liver Fibrosis by Inhibiting the NF-Κb Pathway, Promoting HSC Apoptosis and Suppressing Autophagy. Cell Physiol Biochem 44 (2), 436–446. 10.1159/000485009 29141243

[B2] Chen F. Guo Y. Meng X. Zhang S. (1995). [Identification of Chaihu Guizhi Ganjiang Decoction by Three Dimensional HPLC]. Zhongguo Zhong Yao Za Zhi 20 (4), 223–253. 7646791

[B3] Cui L. H. Li C. X. Zhuo Y. Z. Yang L. Cui M. Q. Zhang S. K. (2019). Saikosaponin D Ameliorates Pancreatic Fibrosis by Inhibiting Autophagy of Pancreatic Stellate Cells via PI3K/Akt/mTOR Pathway. Chemico-biological interactions 300, 18–26. 10.1016/j.cbi.2019.01.005 30611790

[B4] Cui L. Li C. Zhuo Y. Yang L. Cui N. Li Y. (2020). Saikosaponin A Inhibits the Activation of Pancreatic Stellate Cells by Suppressing Autophagy and the NLRP3 Inflammasome via the AMPK/mTOR Pathway. Biomed. Pharmacother. 128, 110216. 10.1016/j.biopha.2020.110216 32497863

[B5] Diakopoulos K. N. Lesina M. Wörmann S. Song L. Aichler M. Schild L. (2015). Impaired Autophagy Induces Chronic Atrophic Pancreatitis in Mice via Sex- and Nutrition-dependent Processes. Gastroenterology 148 (3), 626–638. 10.1053/j.gastro.2014.12.003 25497209

[B6] Ding Y. Zhang Y. He Q. (2017). HE Qing Yong's Experiences in the Application of Chaihu Guizhi Ganjiang Decoction. World J. Integrated Traditional West. Med. 12 (6), 766–768. 10.13935/j.cnki.sjzx.170608

[B7] Endo S. Nakata K. Ohuchida K. Takesue S. Nakayama H. Abe T. (2017). Autophagy Is Required for Activation of Pancreatic Stellate Cells, Associated with Pancreatic Cancer Progression and Promotes Growth of Pancreatic Tumors in Mice. Gastroenterology 152 (6), 1492–1506. 10.1053/j.gastro.2017.01.010 28126348

[B8] Erkan M. Adler G. Apte M. V. Bachem M. G. Buchholz M. Detlefsen S. (2012). StellaTUM: Current Consensus and Discussion on Pancreatic Stellate Cell Research. Gut 61 (2), 172–178. 10.1136/gutjnl-2011-301220 22115911 PMC3245897

[B9] Fan J. Duan L. Wu N. Xu X. Xin J. Jiang S. (2020). Baicalin Ameliorates Pancreatic Fibrosis by Inhibiting the Activation of Pancreatic Stellate Cells in Mice with Chronic Pancreatitis. Front. Pharmacol. 11, 607133. 10.3389/fphar.2020.607133 33536916 PMC7848203

[B10] Gonzalez C. D. Lee M.-S. Marchetti P. Pietropaolo M. Towns R. Vaccaro M. I. (2011). The Emerging Role of Autophagy in the Pathophysiology of Diabetes Mellitus. Autophagy 7 (1), 2–11. 10.4161/auto.7.1.13044 20935516 PMC3359481

[B11] Hart P. A. Conwell D. L. (2020). Chronic Pancreatitis: Managing a Difficult Disease. Am. J. Gastroenterol. 115 (1), 49–55. 10.14309/ajg.0000000000000421 31764092 PMC6940526

[B12] Hong Z.-P. Wang L.-G. Wang H.-J. Ye W.-F. Wang X.-Z. (2018). Wogonin Exacerbates the Cytotoxic Effect of Oxaliplatin by Inducing Nitrosative Stress and Autophagy in Human Gastric Cancer Cells. Phytomedicine 39, 168–175. 10.1016/j.phymed.2017.12.019 29433678

[B13] Hosseinzadeh A. Javad-Moosavi S. A. Reiter R. J. Yarahmadi R. Ghaznavi H. Mehrzadi S. (2018). Oxidative/nitrosative Stress, Autophagy and Apoptosis as Therapeutic Targets of Melatonin in Idiopathic Pulmonary Fibrosis. Expert Opin. Ther. Targets 22 (12), 1049–1061. 10.1080/14728222.2018.1541318 30445883

[B14] Inoue M. Ino Y. Gibo J. Ito T. Hisano T. Arita Y. (2002). The Role of Monocyte Chemoattractant Protein-1 in Experimental Chronic Pancreatitis Model Induced by Dibutyltin Dichloride in Rats. Pancreas 25 (4), e64–e70. 10.1097/00006676-200211000-00023 12409843

[B15] Itoh T. Michijiri S. Murai S. Saito H. Saito H. Itsukaichi O. (1996). Effects of Chaihu-Guizhi-Ganjiang-Tang on the Levels of Monoamines and Their Related Substances, and Acetylcholine in Discrete Brain Regions of Mice. Am. J. Chin. Med. 24 (1), 53–64. 10.1142/s0192415x9600008610.1142/s0192415x9600027x 8739182

[B16] Kirkegård J. Mortensen F. V. Cronin-Fenton D. (2017). Chronic Pancreatitis and Pancreatic Cancer Risk: A Systematic Review and Meta-Analysis. Am. J. Gastroenterol. 112 (9), 1366–1372. 10.1038/ajg.2017.218 28762376

[B17] Li C.-X. Cui L.-H. Zhuo Y.-Z. Hu J.-G. Cui N.-Q. Zhang S.-K. (2018a). Inhibiting Autophagy Promotes Collagen Degradation by Regulating Matrix Metalloproteinases in Pancreatic Stellate Cells. Life Sci. 208, 276–283. 10.1016/j.lfs.2018.07.049 30056017

[B18] Li L. Wang G. Hu J.-S. Zhang G.-Q. Chen H.-Z. Yuan Y. (2018b). RB1CC1-enhanced Autophagy Facilitates PSCs Activation and Pancreatic Fibrogenesis in Chronic Pancreatitis. Cell Death Dis 9 (10), 952. 10.1038/s41419-018-0980-4 30237496 PMC6147947

[B19] Li Z. Cheng J. Liu J. (2020). Baicalin Protects Human OA Chondrocytes against IL-1β-Induced Apoptosis and ECM Degradation by Activating Autophagy via MiR-766-3p/AIFM1 Axis. Drug. Des. Devil. Ther. Vol. 14, 2645–2655. 10.2147/dddt.s255823 PMC7353997 32753846

[B20] Liu C. Li S. Zhang Q. Guo F. Tong M. Martinez M. F. Y. M. (2019). Emerging Role of Chinese Herbal Medicines in the Treatment of Pancreatic Fibrosis. Am. J. Chin. Med. 47 (4), 709–726. 10.1142/s0192415x1950037x 31091974

[B21] Ma J. Chen L. (2017). Experience of Treating Pancreatic Cancer with Chaihu Guizhi Ganjiang Decoction. Cardiovasc. Dis. J. integrated traditional Chin. West. Med. 5 (26), 195–196. 10.16282/cnki.cn11-9336/r

[B22] Masamune A. Kikuta K. Suzuki N. Satoh M. Satoh K. Shimosegawa T. (2004). A C-Jun NH2-terminal Kinase Inhibitor SP600125 (Anthra[1,9-cd]pyrazole-6 (2H)-One) Blocks Activation of Pancreatic Stellate Cells. J. Pharmacol. Exp. Ther. 310 (2), 520–527. 10.1124/jpet.104.067280 15056726

[B23] McCarroll J. A. Phillips P. A. Park S. Doherty E. Pirola R. C. Wilson J. S. (2003). Pancreatic Stellate Cell Activation by Ethanol and Acetaldehyde: Is it Mediated by the Mitogen-Activated Protein Kinase Signaling Pathway? Pancreas 27 (2), 150–160. 10.1097/00006676-200308000-00008 12883264

[B24] McCarroll J. A. Phillips P. Santucci N. Pirola R. Wilson J. Apte M. (2006). Vitamin A Inhibits Pancreatic Stellate Cell Activation: Implications for Treatment of Pancreatic Fibrosis. Gut 55 (1), 79–89. 10.1136/gut.2005.064543 16043492 PMC1856372

[B25] Niina Y. Ito T. Oono T. Nakamura T. Fujimori N. Igarashi H. (2014). A Sustained Prostacyclin Analog, ONO-1301, Attenuates Pancreatic Fibrosis in Experimental Chronic Pancreatitis Induced by Dibutyltin Dichloride in Rats. Pancreatology 14 (3), 201–210. 10.1016/j.pan.2014.02.009 24854616

[B26] Omary M. B. Lugea A. Lowe A. W. Pandol S. J. (2007). The Pancreatic Stellate Cell: a star on the Rise in Pancreatic Diseases. J. Clin. Invest. 117 (1), 50–59. 10.1172/jci30082 17200706 PMC1716214

[B27] Qu L. Chen C. He W. Chen Y. Li Y. Wen Y. (2019). Glycyrrhizic Acid Ameliorates LPS-Induced Acute Lung Injury by Regulating Autophagy through the PI3K/AKT/mTOR Pathway. Am. J. Transl Res. 11 (4), 2042–2055. 31105816 PMC6511780

[B28] Ramakrishnan P. Loh W. M. Gopinath S. C. B. Bonam S. R. Fareez I. M. Mac Guad R. (2020). Selective Phytochemicals Targeting Pancreatic Stellate Cells as New Anti-fibrotic Agents for Chronic Pancreatitis and Pancreatic Cancer. Acta. Pharmaceutica. Sinica. B. 10 (3), 399–413. 10.1016/j.apsb.2019.11.008 32140388 PMC7049637

[B29] Sousa C. M. Biancur D. E. Wang X. Halbrook C. J. Sherman M. H. Zhang L. (2016). Pancreatic Stellate Cells Support Tumour Metabolism through Autophagic Alanine Secretion. Nature 536 (7617), 479–483. 10.1038/nature19084 27509858 PMC5228623

[B30] Tian Y. Jia S.-X. Shi J. Gong G.-Y. Yu J.-W. Niu Y. (2019). Polyphyllin I Induces Apoptosis and Autophagy via Modulating JNK and mTOR Pathways in Human Acute Myeloid Leukemia Cells. Chemico-biological interactions 311, 108793. 10.1016/j.cbi.2019.108793 31421117

[B31] Tu Y. Gu L. Chen D. Wu W. Liu H. Hu H. (2017). Rhein Inhibits Autophagy in Rat Renal Tubular Cells by Regulation of AMPK/mTOR Signaling. Sci. Rep. 7, 43790. 10.1038/srep43790 28252052 PMC5333140

[B32] Wei F. Jiang X. Gao H.-Y. Gao S.-H. (2017). Liquiritin Induces Apoptosis and Autophagy in Cisplatin (DDP)-resistant Gastric Cancer Cells *In Vitro* and Xenograft Nude Mice *In Vivo* . Int. J. Oncol. 51 (5), 1383–1394. 10.3892/ijo.2017.4134 29048624 PMC5642394

[B33] Witt H. Apte M. V. Keim V. Wilson J. S. (2007). Chronic Pancreatitis: Challenges and Advances in Pathogenesis, Genetics, Diagnosis, and Therapy. Gastroenterology 132 (4), 1557–1573. 10.1053/j.gastro.2007.03.001 17466744

[B34] Xu X.-F. Liu F. Xin J.-Q. Fan J.-W. Wu N. Zhu L.-J. (2018). Respective Roles of the Mitogen-Activated Protein Kinase (MAPK) Family Members in Pancreatic Stellate Cell Activation Induced by Transforming Growth Factor-Β1 (TGF-Β1). Biochem. biophysical Res. Commun. 501 (2), 365–373. 10.1016/j.bbrc.2018.04.176 29705706

[B35] Xue R. Yang J. Wu J. Meng Q. Hao J. (2017). Coenzyme Q10 Inhibits the Activation of Pancreatic Stellate Cells through PI3K/AKT/mTOR Signaling Pathway. Oncotarget 8 (54), 92300–92311. 10.18632/oncotarget.21247 29190916 PMC5696182

[B36] Yang X. Peng W. Yue X. (2009). Syndrome Differentiation and Treatment of Taiyang Disease in Shanghan Lun. J. Chin. Integr. Med. 7 (2), 171–174. 10.3736/jcim20090215 19216863

[B37] Yu C. Li W.-b. Liu J.-b. Lu J.-w. Feng J.-f. (2018). Autophagy: Novel Applications of Nonsteroidal Anti-inflammatory Drugs for Primary Cancer. Cancer Med. 7 (2), 471–484. 10.1002/cam4.1287 29282893 PMC5806108

[B38] Yue J. López J. M. (2020). Understanding MAPK Signaling Pathways in Apoptosis. Int. J. Mol. Sci. 21 (7), 2346. 10.3390/ijms21072346 PMC7177758 32231094

[B39] Zeng X.-P. Wang L.-J. Guo H.-L. He L. Bi Y.-W. Xu Z.-L. (2019). Dasatinib Ameliorates Chronic Pancreatitis Induced by Caerulein via Anti-fibrotic and Anti-inflammatory Mechanism. Pharmacol. Res. 147, 104357. 10.1016/j.phrs.2019.104357 31356863

[B40] Zhang S.-k. Cui N.-Q. Zhuo Y.-z. Li D.-h. Liu J.-h. (2013). Modified Xiaochaihu Decoction Prevents the Progression of Chronic Pancreatitis in Rats Possibly by Inhibiting Transforming Growth Factor-β1/Sma- and Mad-Related Proteins Signaling Pathway. Chin. J. Integr. Med. 19 (12), 935–939. 10.1007/s11655-013-1656-7 24307314

[B41] Zhang L. Wang H. Cong Z. Xu J. Zhu J. Ji X. (2014). Wogonoside Induces Autophagy-Related Apoptosis in Human Glioblastoma Cells. Oncol. Rep. 32 (3), 1179–1187. 10.3892/or.2014.3294 24970553

[B42] Zhang S.-k. Cui N.-Q. Zhuo Y.-z. Hu J.-g. Liu J.-h. Li D.-h. (2017). Modified Xiaochaihu Decoction Promotes Collagen Degradation and Inhibits Pancreatic Fibrosis in Chronic Pancreatitis Rats. Chin. J. Integr. Med. 26, 599–603. 10.1007/s11655-017-2413-0 29181733

[B43] Zhao S.-M. Li H. Guo C. Shen L. (2010). Cardiac Fibrosis in Diabetic Rats: Regulation and Mechanism of Activation of the PPARγ Signal Pathway. Chin. J. Physiol. 53 (4), 262–267. 10.4077/cjp.2010.amk076 21793336

