# Supplementary figures and images for: Chaihu Guizhi Ganjiang Decoction Ameliorates Pancreatic Fibrosis via JNK/mTOR Signaling Pathway
Source: Front Pharmacol. 2021 Jun 10;12:679557. doi: 10.3389/fphar.2021.679557 (PMC8223066; doi:10.3389/fphar.2021.679557)

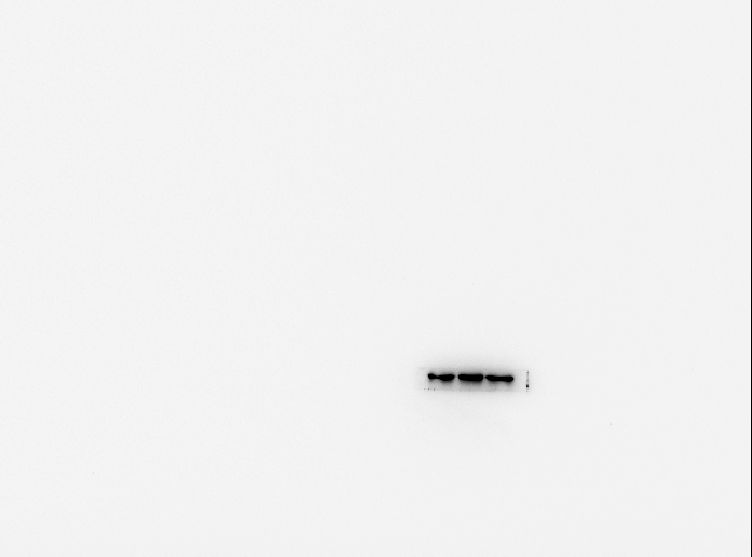

Supplement: Supplementary file 1 [file datasheet1.zip › WB original pictures/Fig.3D-a-SMA.jpg]

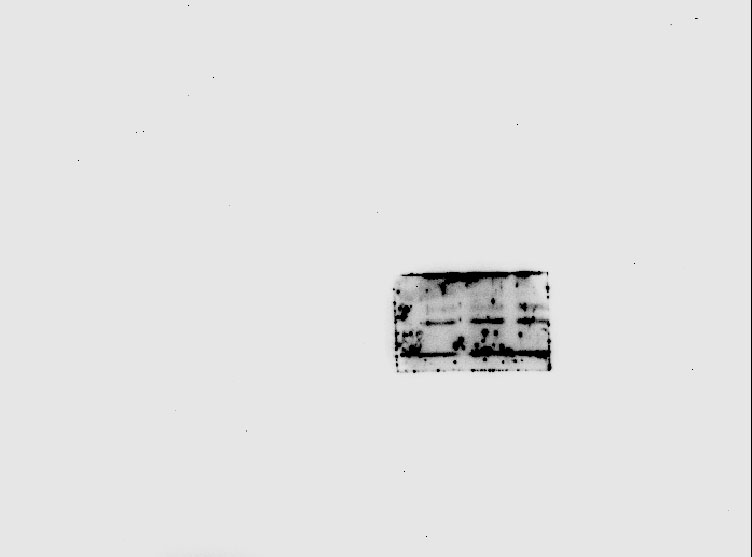

Supplement: Supplementary file 1 [file datasheet1.zip › WB original pictures/Fig.3D-COLI.jpg]

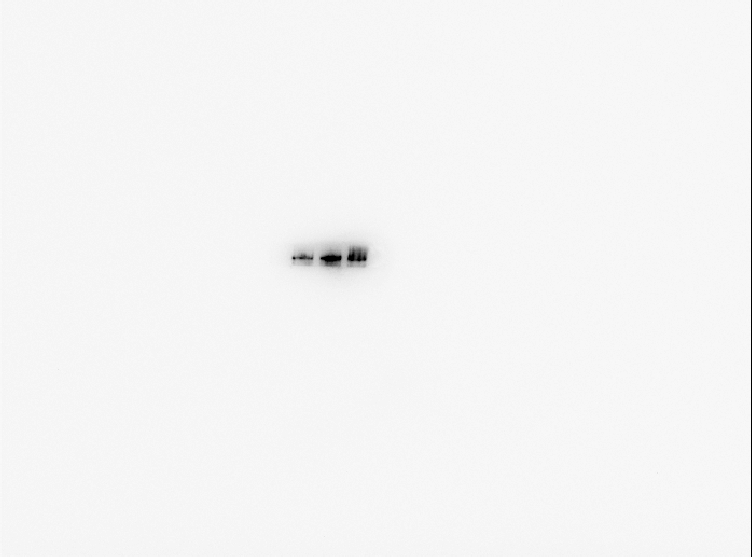

Supplement: Supplementary file 1 [file datasheet1.zip › WB original pictures/Fig.3D-FN.jpg]

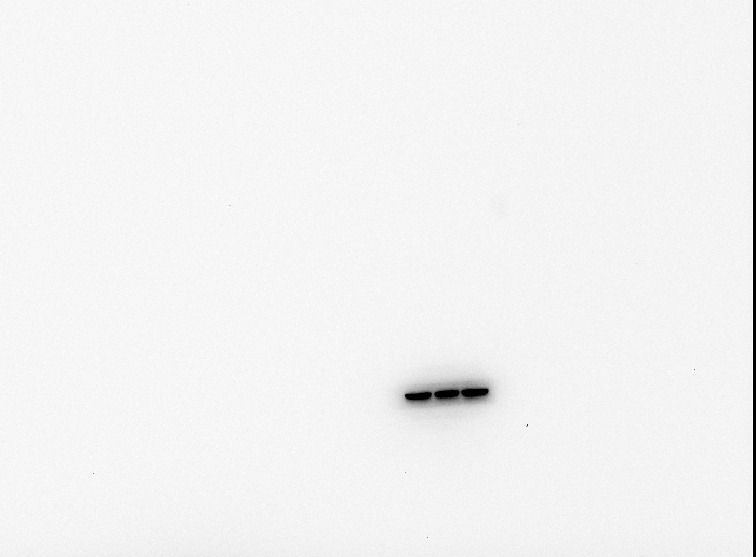

Supplement: Supplementary file 1 [file datasheet1.zip › WB original pictures/Fig.3D-GAPDH.jpg]

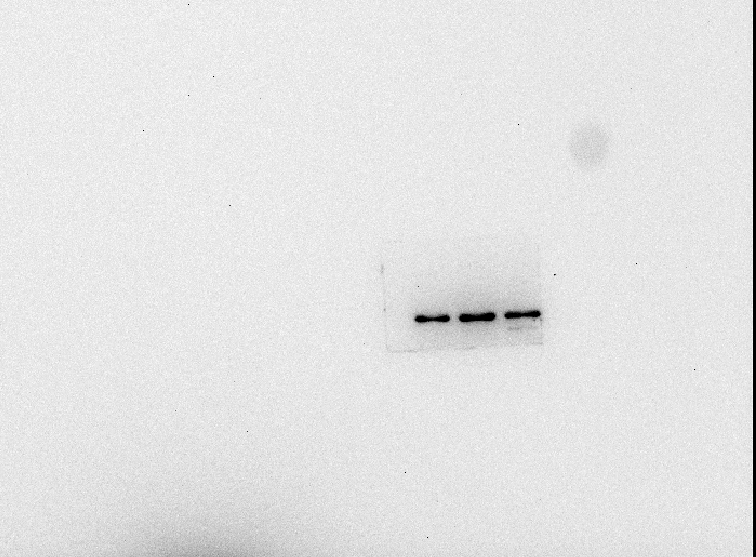

Supplement: Supplementary file 1 [file datasheet1.zip › WB original pictures/Fig.4C-Atg5.jpg]

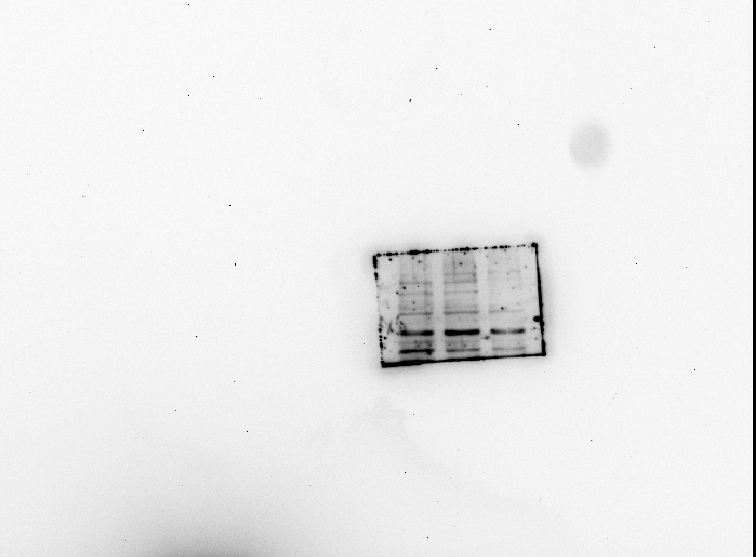

Supplement: Supplementary file 1 [file datasheet1.zip › WB original pictures/Fig.4C-Beclin-1.jpg]

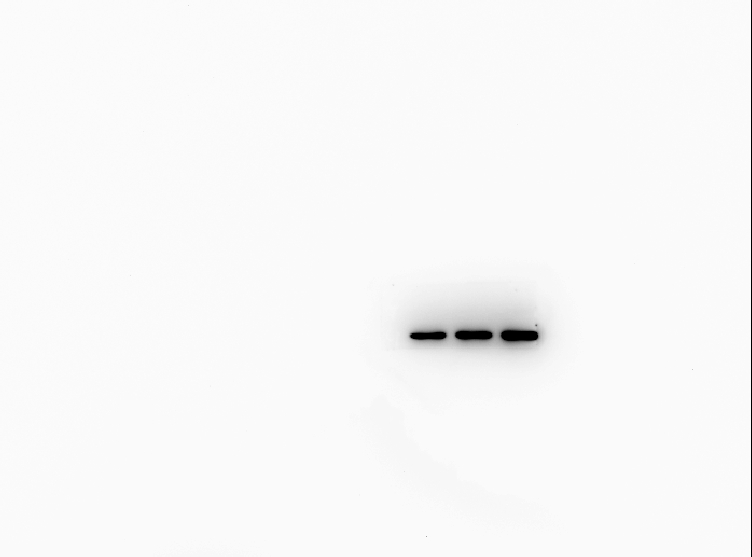

Supplement: Supplementary file 1 [file datasheet1.zip › WB original pictures/Fig.4C-GAPDH.jpg]

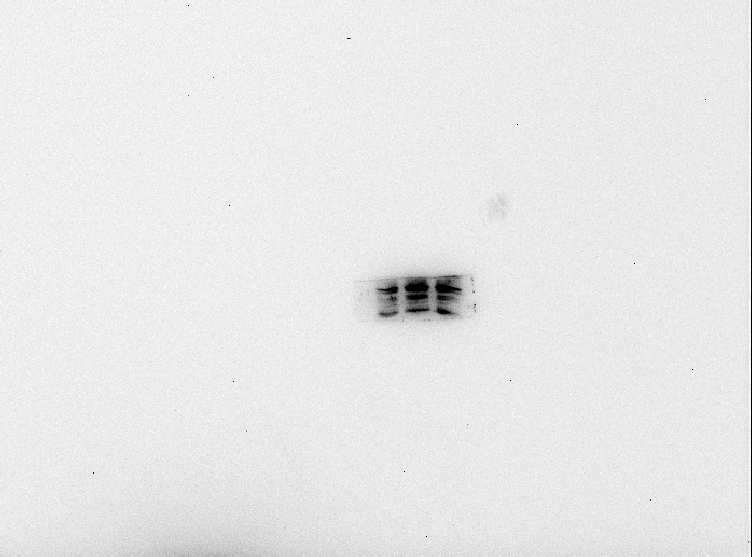

Supplement: Supplementary file 1 [file datasheet1.zip › WB original pictures/Fig.4C-LC3B.jpg]

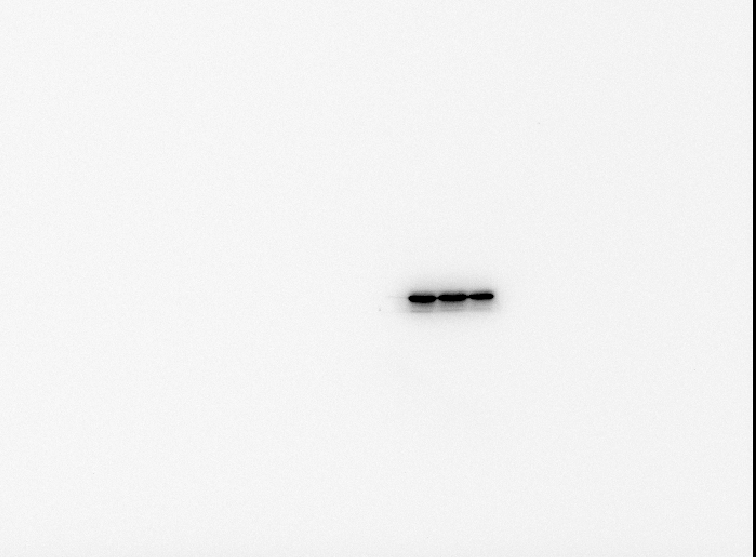

Supplement: Supplementary file 1 [file datasheet1.zip › WB original pictures/Fig.4D-GAPDH.jpg]

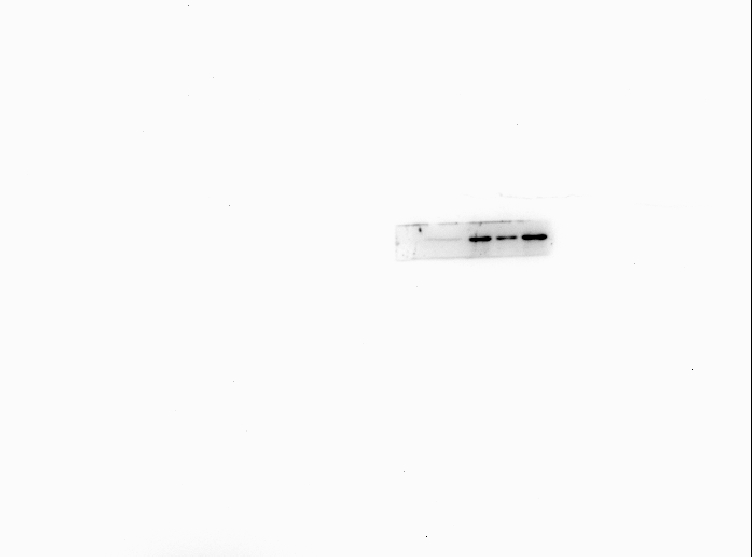

Supplement: Supplementary file 1 [file datasheet1.zip › WB original pictures/Fig.4D-mTOR.jpg]

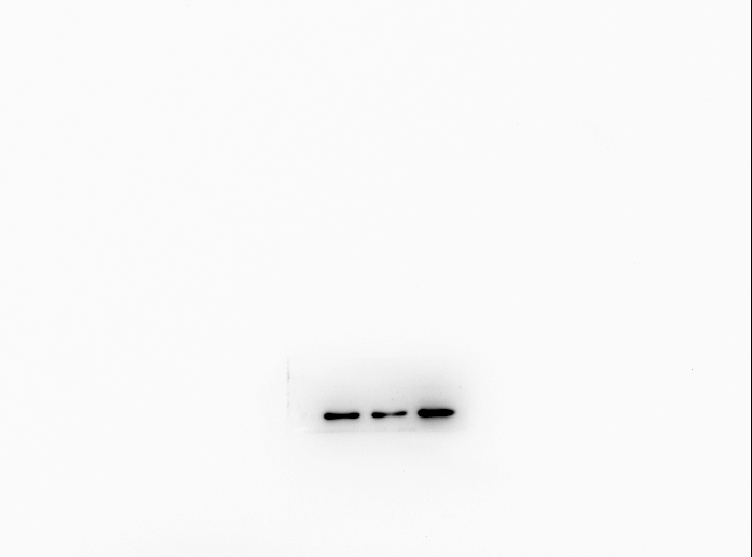

Supplement: Supplementary file 1 [file datasheet1.zip › WB original pictures/Fig.4D-p-mTOR.jpg]

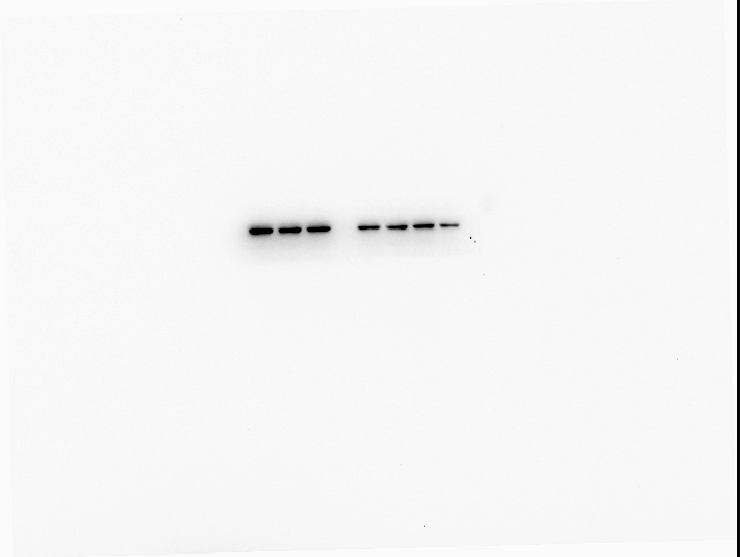

Supplement: Supplementary file 1 [file datasheet1.zip › WB original pictures/Fig.5B-a-SMA.jpg]

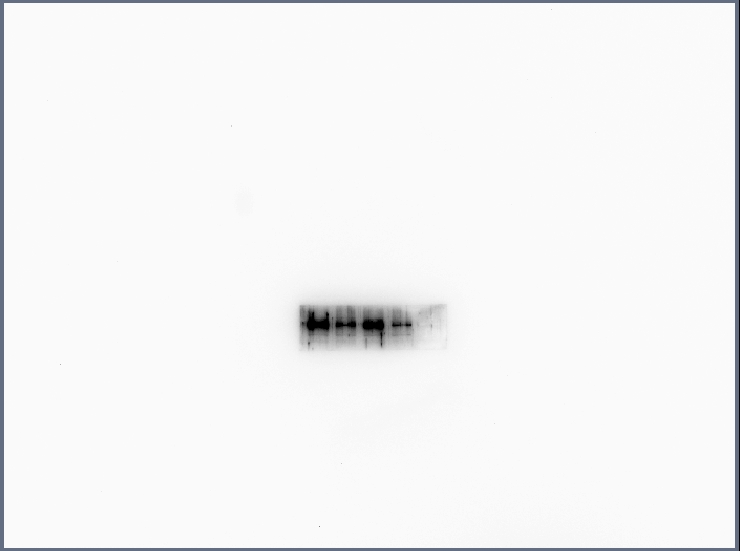

Supplement: Supplementary file 1 [file datasheet1.zip › WB original pictures/Fig.5B-COL-1.jpg]

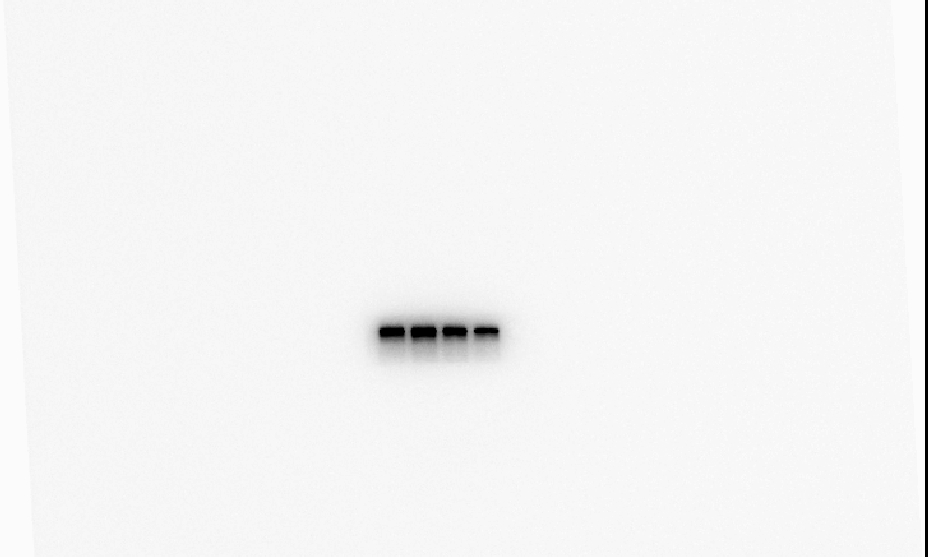

Supplement: Supplementary file 1 [file datasheet1.zip › WB original pictures/Fig.5B-FN.jpg]

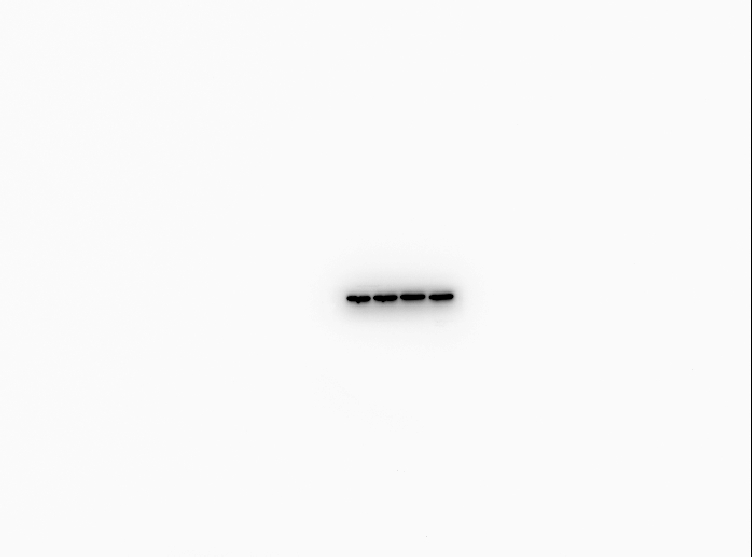

Supplement: Supplementary file 1 [file datasheet1.zip › WB original pictures/Fig.5B-GAPDH.jpg]

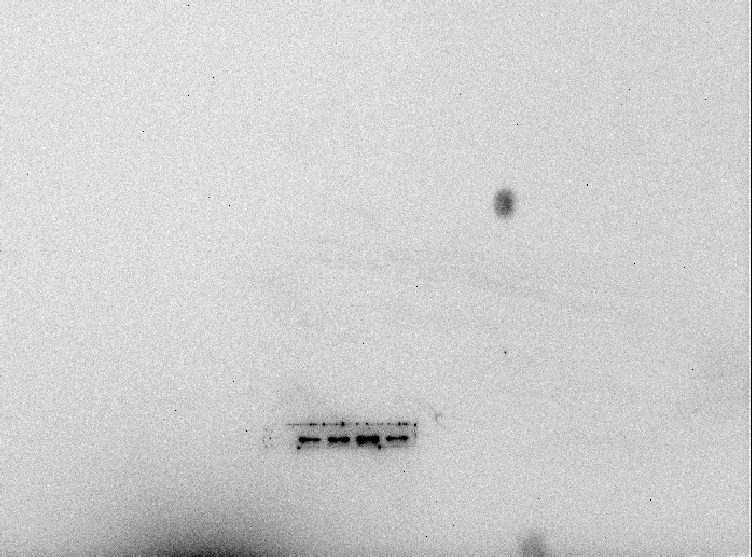

Supplement: Supplementary file 1 [file datasheet1.zip › WB original pictures/Fig.5E-Atg5.jpg]

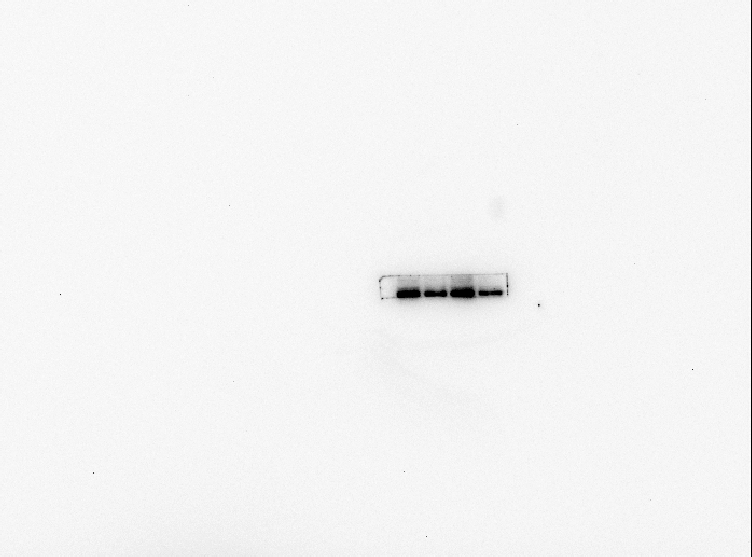

Supplement: Supplementary file 1 [file datasheet1.zip › WB original pictures/Fig.5E-Beclin-1.jpg]

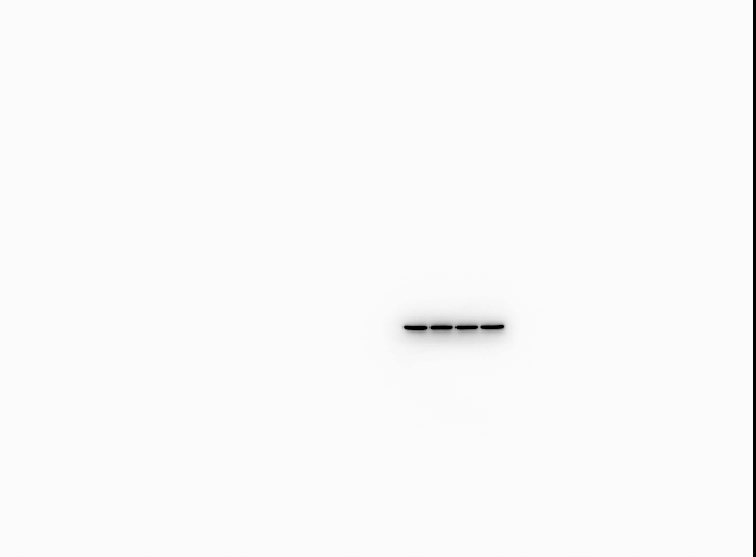

Supplement: Supplementary file 1 [file datasheet1.zip › WB original pictures/Fig.5E-GAPDH.jpg]

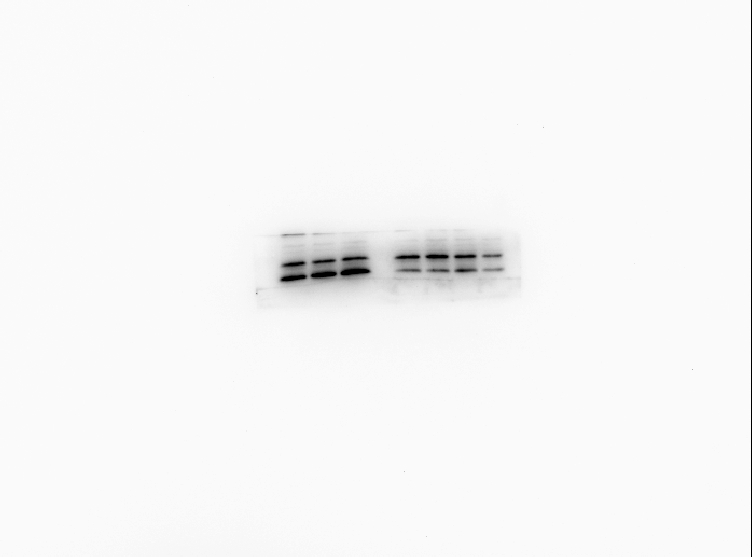

Supplement: Supplementary file 1 [file datasheet1.zip › WB original pictures/Fig.5E-LC3B.jpg]

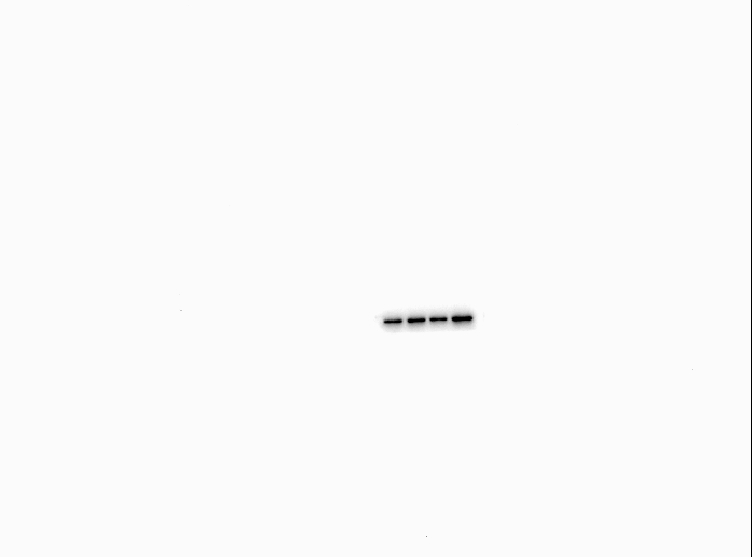

Supplement: Supplementary file 1 [file datasheet1.zip › WB original pictures/Fig.5E-P62.jpg]

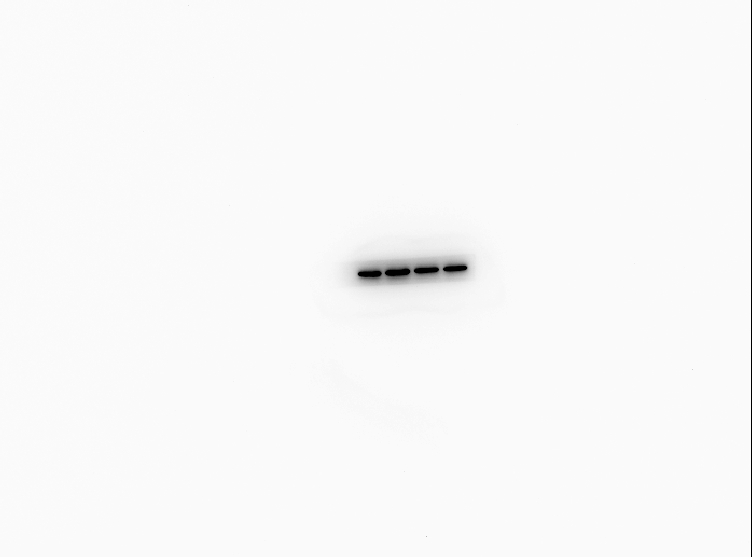

Supplement: Supplementary file 1 [file datasheet1.zip › WB original pictures/Fig.6A-GAPDH.jpg]

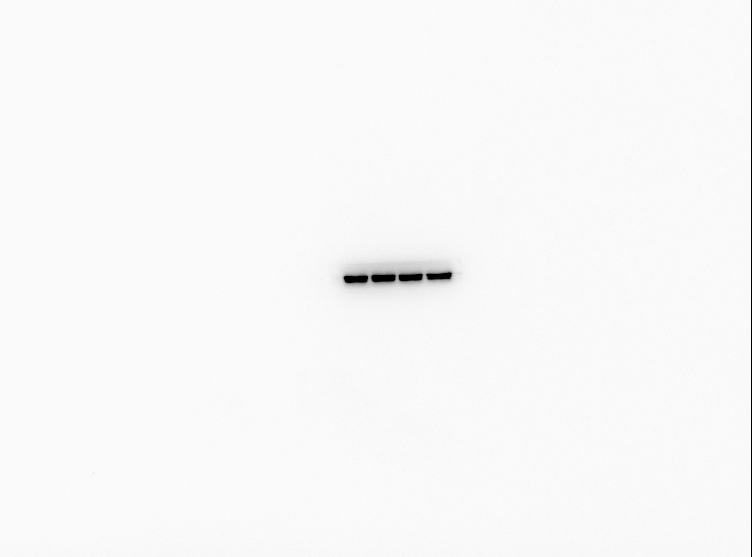

Supplement: Supplementary file 1 [file datasheet1.zip › WB original pictures/Fig.6A-p-AKT.jpg]

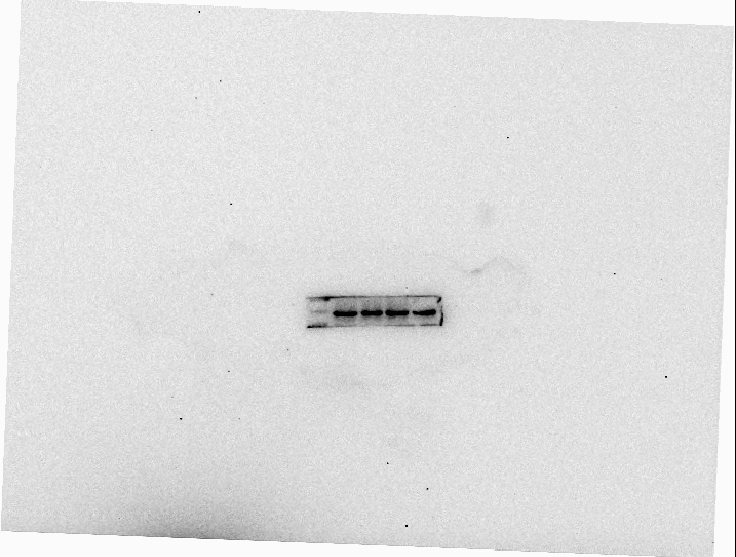

Supplement: Supplementary file 1 [file datasheet1.zip › WB original pictures/Fig.6A-p-AMPK.jpg]

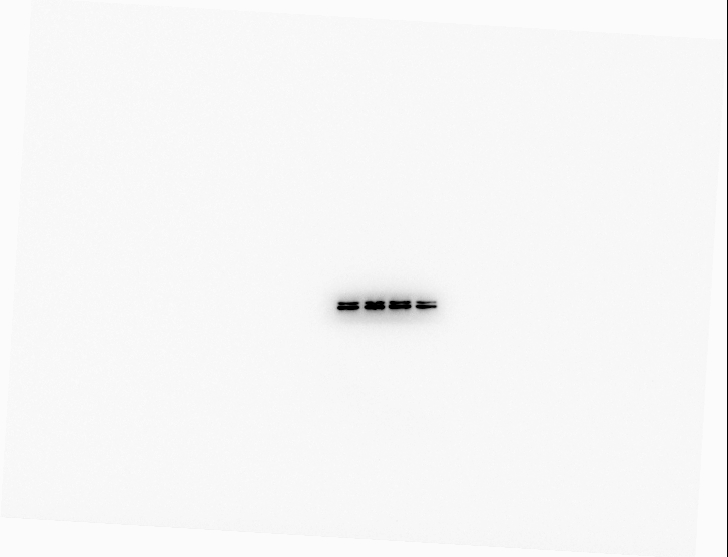

Supplement: Supplementary file 1 [file datasheet1.zip › WB original pictures/Fig.6A-p-ERK.jpg]

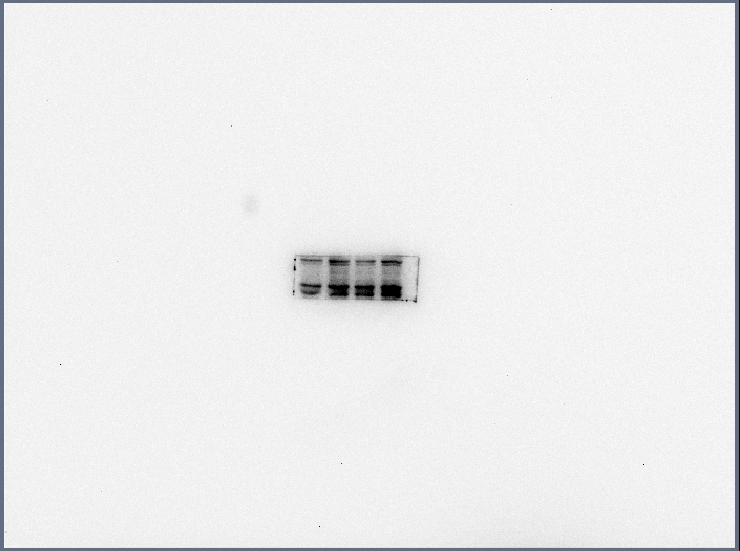

Supplement: Supplementary file 1 [file datasheet1.zip › WB original pictures/Fig.6A-p-JNK.jpg]

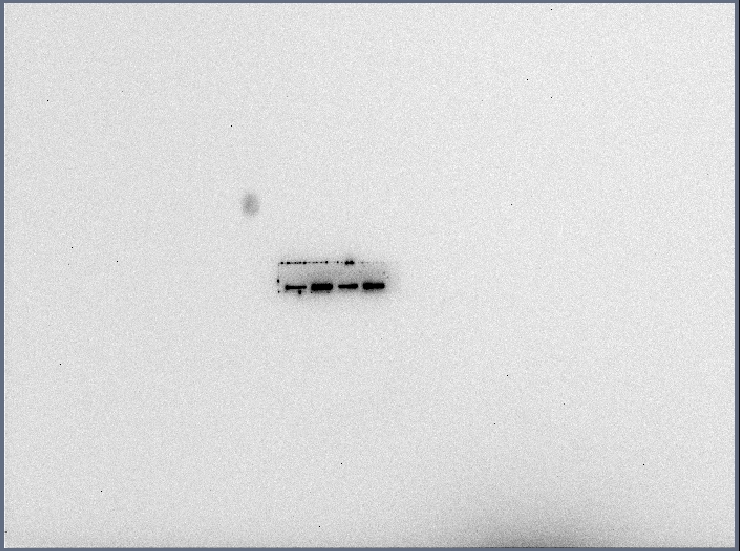

Supplement: Supplementary file 1 [file datasheet1.zip › WB original pictures/Fig.6A-p-mTOR.jpg]

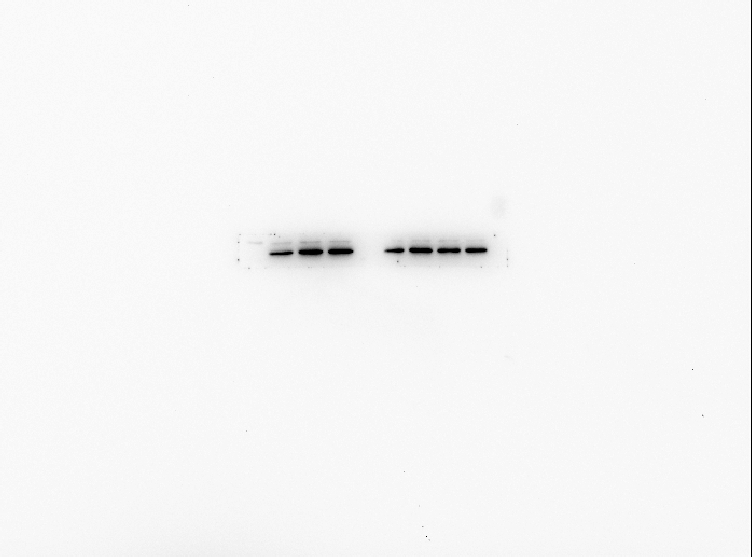

Supplement: Supplementary file 1 [file datasheet1.zip › WB original pictures/Fig.6A-p-P38.jpg]

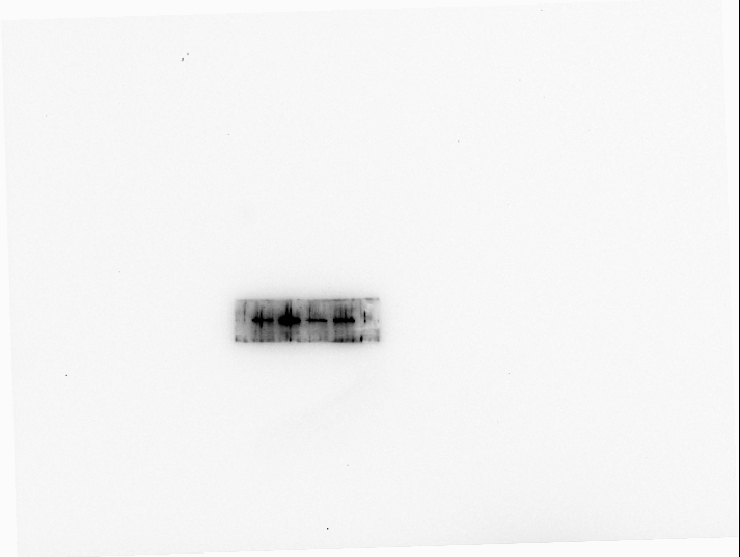

Supplement: Supplementary file 1 [file datasheet1.zip › WB original pictures/Fig.6B-COL-1.jpg]

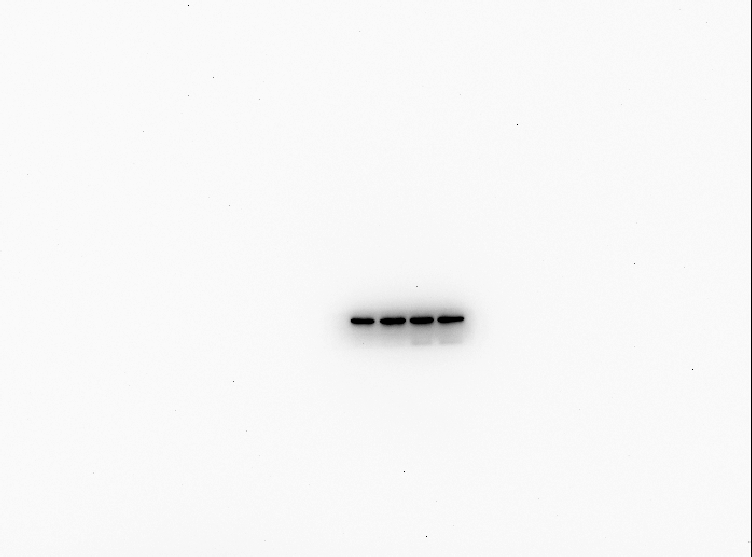

Supplement: Supplementary file 1 [file datasheet1.zip › WB original pictures/Fig.6B-GAPDH.jpg]

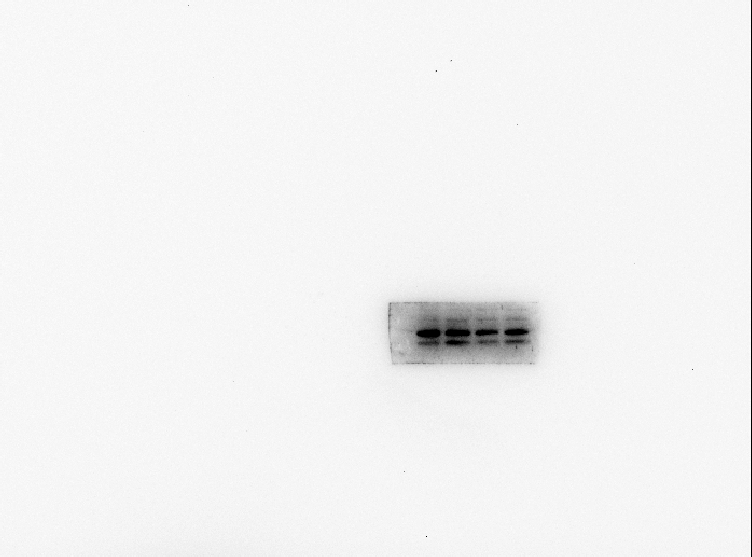

Supplement: Supplementary file 1 [file datasheet1.zip › WB original pictures/Fig.6B-LC3B.jpg]

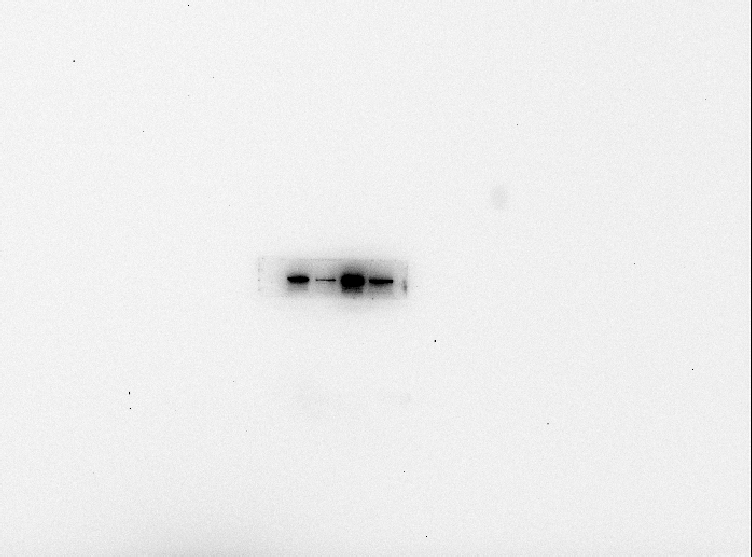

Supplement: Supplementary file 1 [file datasheet1.zip › WB original pictures/Fig.6B-p-mTOR.jpg]

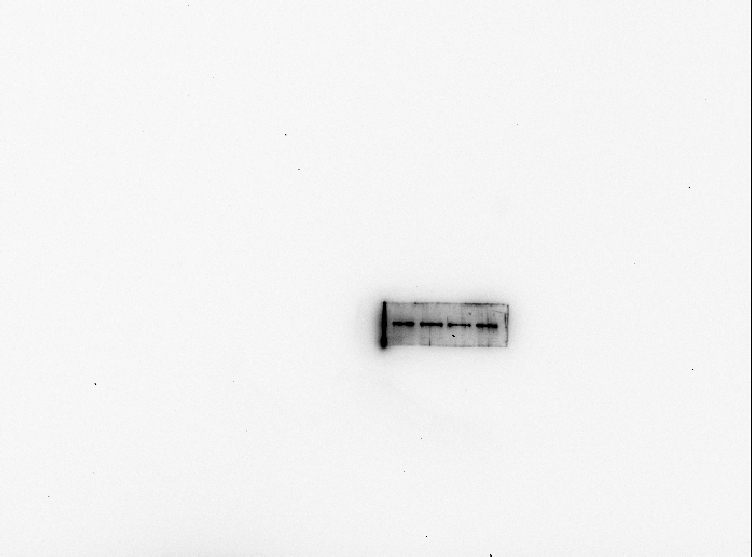

Supplement: Supplementary file 1 [file datasheet1.zip › WB original pictures/Fig.6C-COL-1.jpg]

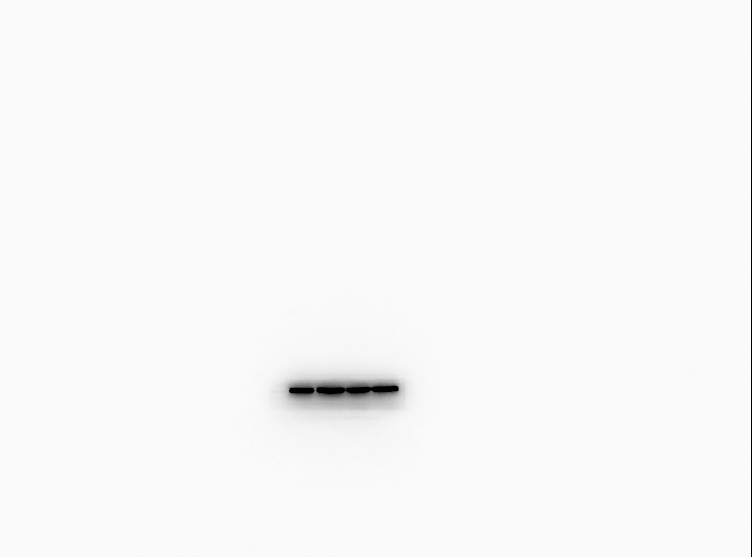

Supplement: Supplementary file 1 [file datasheet1.zip › WB original pictures/Fig.6C-GAPDH.jpg]

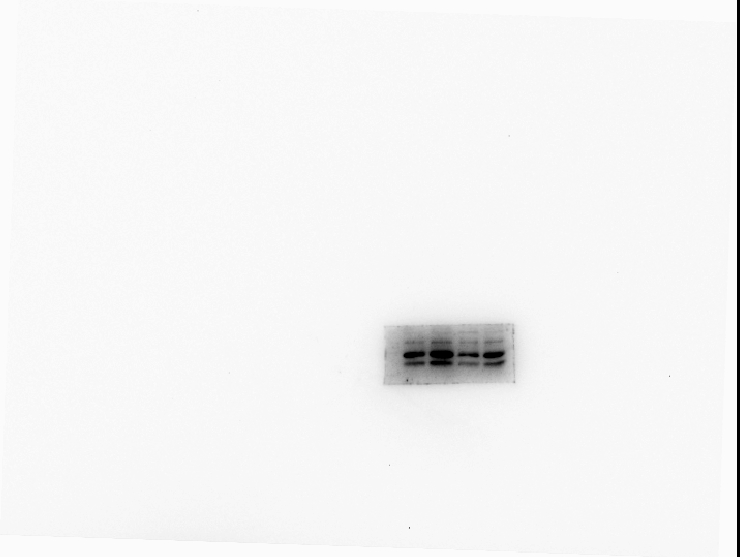

Supplement: Supplementary file 1 [file datasheet1.zip › WB original pictures/Fig.6C-LC3B.jpg]

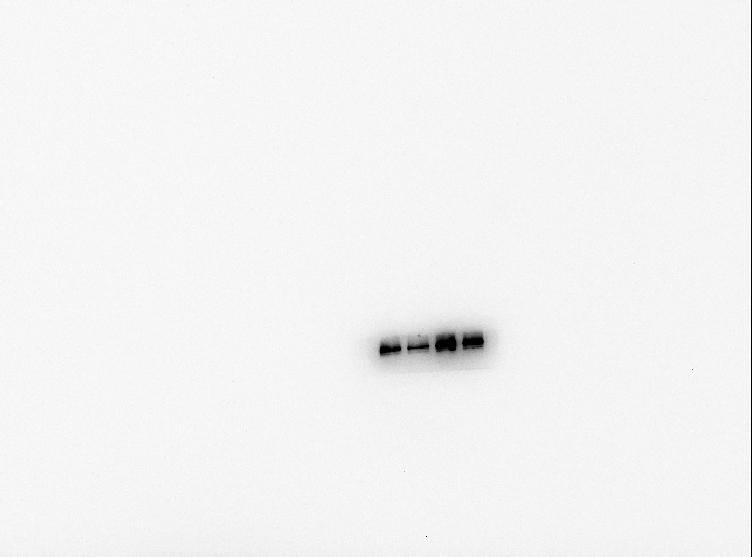

Supplement: Supplementary file 1 [file datasheet1.zip › WB original pictures/Fig.6C-p-JNK.jpg]
